# Differential Trends in the Codon Usage Patterns in HIV-1 Genes

**DOI:** 10.1371/journal.pone.0028889

**Published:** 2011-12-22

**Authors:** Aridaman Pandit, Somdatta Sinha

**Affiliations:** 1 Mathematical Modeling and Computational Biology Group, Centre for Cellular & Molecular Biology (CSIR), Hyderabad, Andhra Pradesh, India; 2 Indian Institute of Science Education and Research Mohali, Mohali, Punjab, India; University of Toronto, Canada

## Abstract

Host-pathogen interactions underlie one of the most complex evolutionary phenomena resulting in continual adaptive genetic changes, where pathogens exploit the host's molecular resources for growth and survival, while hosts try to eliminate the pathogen. Deciphering the molecular basis of host–pathogen interactions is useful in understanding the factors governing pathogen evolution and disease propagation. In host-pathogen context, a balance between mutation, selection, and genetic drift is known to maintain codon bias in both organisms. Studies revealing determinants of the bias and its dynamics are central to the understanding of host-pathogen evolution. We considered the Human Immunodeficiency Virus (HIV) type 1 and its human host to search for evolutionary signatures in the viral genome. Positive selection is known to dominate intra-host evolution of HIV-1, whereas high genetic variability underlies the belief that neutral processes drive inter-host differences. In this study, we analyze the codon usage patterns of HIV-1 genomes across all subtypes and clades sequenced over a period of 23 years. We show presence of unique temporal correlations in the codon bias of three HIV-1 genes illustrating differential adaptation of the HIV-1 genes towards the host preferred codons. Our results point towards gene-specific translational selection to be an important force driving the evolution of HIV-1 at the population level.

## Introduction

Host-pathogen interactions underlie one of the most complex and interesting evolutionary phenomena, where reproductive fitness of one species is affected by its reciprocal interactions with the other. The pathogens engage in a quest to maximize their replicative fitness governed by several factors like infectivity, virulence, the ability to hide within the host, etc. Host-pathogen interactions result in continual adaptive genetic changes or “arms race”, where pathogens develop means to exploit most out of the host's molecular resources for their growth and survival, while hosts try to render pathogenic attacks futile and eliminate the pathogen [1]. Hosts, like human, have acquired several artilleries that induce complex defense response against the pathogens and this response may vary from individual to individual. Pathogens, on the other hand, mutate to evade the host defense mechanisms. The host-pathogen interaction, thus, is a major selective force that drives the evolution of host and pathogen both within an individual host and at the population level [2], [3]. Deciphering the molecular basis of these interactions is useful in understanding the factors governing pathogen evolution and disease propagation.

Genetic code is degenerate as multiple codons code for a single amino acid. Most organisms exhibit differences in base composition and significant codon bias (unequal usage of synonymous codons). Generally, mutations leading to change of amino acids are studied as a measure of selection. Synonymous mutations can change the base composition of genes without altering the corresponding proteins. Intuitively, synonymous mutations appear to be “neutral” or “near-neutral” in their effects, however, their evolutionary consequences are being increasingly understood [4]–[8]. Studies show that codon bias and synonymous mutations are under weak selection, driving evolution in various organisms [9]–[11]. Genes that are enriched for the preferred codons are known to have higher translational efficiency [12]–[13]. It has been shown in other host-pathogen systems, such as bacteria-bacteriophages, that long-term co-evolution has resulted in some genes of bacteriophages being enriched in the codons preferred by their respective bacterial hosts [14]. In contrast, several RNA viruses show low association with their hosts in both base composition and codon usage [15]. A balance between selection, mutation, and genetic drift maintains codon bias in host and pathogens. Thus, studies revealing determinants of the bias and its dynamics are central to the understanding of host-pathogen evolution [1], [16]. With their short generation times, pathogens like viruses offer possibilities to study evolution over observable time scales, as has been shown for studies with HIV within patients [17]–[19].

Exploitation of the extensive molecular and genomic data available on the host and pathogen genomes can be of great use in studying the determinants of their interactions and evolution. In an attempt to search for such evidences using genomic data, we undertook studies on a pathogen, which fit the following criteria – high reproductive rate, obligate on a single host, availability of large number of gene/genome sequences, and most importantly, availability of genomic data over many years from a large population. In this paper, we examine the codon usage pattern as a signature of pathogen genome evolution in host-pathogen interactions, for the Human Immunodeficiency Virus type 1 (HIV-1), in relation to its human host.

HIV-1, a highly infectious retrovirus that crossed host from chimpanzees to humans, is the causative agent of the global pandemic of Acquired Immunodeficiency Syndrome (AIDS) responsible for more than 25 million deaths [20], [21]. HIV-1 is capable of undergoing rapid sequence level changes to evade the host's defense machinery, which is partially a result of strong immune selection pressure levied by the host. High rates of mutation, frequent recombination events, and multiple cross-species transmissions have led to enormous genetic diversity in HIV-1. This has been a major impediment to the development of a foolproof strategy for countering the disease [22], [23]. Inability of the host's immune system to control HIV-1 spread and lack of cure, both have added to the success story of HIV-1 as a pathogen.

HIV-1 uses the host's translational machinery to translate their own mRNA. Thus, HIV-1 is a translational parasite on humans. HIV-1 genome comprises of nine genes, which can be divided into two classes based on their function: *structural genes* (*env*, *gag*, and *pol*) that form the essential components of the virus particle [18], [19]; and *regulatory genes* (*nef*, *rev*, *tat*, *vif*, *vpr*, and *vpu*). Of these, the regulatory genes *rev* and *tat* are mandatory [24]–[26] and the remaining four genes are termed “accessory”, as they are required for efficient replication of the virus, but have been shown not to be mandatory for survival *in vitro* [27]–[29]. The importance of the regulatory and accessory genes is being understood increasingly. Most evolutionary arguments for HIV-1 are based on studies with the genes coding for structural proteins (*env*, *gag*, and *pol*), because of their therapeutic relevance. Phylogenetic analysis of *env* and *gag* sequences from longitudinal studies in patients over time has been indicative of strong selection operating on the virus to form escape mutants inside the host [17], [30].

HIV-1 has an A-rich genome, attributed to ‘G’ to ‘A’ hypermutations by the host APOBEC gene-family [3] and to the Reverse Transcriptase enzyme [31], while the host human genome is known to be GC-rich in coding regions. Studies on *env* gene in patients have shown that codon usage became more homogeneous and more correlated with host human's codon bias over time [32]. At the population level also, various studies have demonstrated similar selection of functionally important sites in different HIV-1 subtypes, arguing for presence of positive selection [33], [34]. Contrary to these studies, genetic/mutational drift has also been shown to drive evolution of HIV-1 in inter-host transmission, as immune selection changes with change in individual host [17], [35]. Given these opposing results, a clear understanding of the mechanisms leading to host-specific adaptation of the virus, both within a particular individual and at the population level, can provide plausible targets for drug therapy and help to develop effective vaccines.

Using HIV-1 as a model system, we address the question: do pathogens progressively adapt towards host codon bias to exploit the host's molecular machinery effectively? To investigate this, we performed a large-scale analysis of the codon usage patterns of the nine genes of HIV-1 from 1357 whole genomes collected over a period of 23 years. We show differential temporal correlation in codon usage patterns among the HIV-1 genes towards the human codon usage pattern, indicating an important functional role of selection to improve translational efficiency in host-pathogen interactions. This study also highlights the significance of synonymous mutations in host-pathogen co-evolution.

## Materials and Methods

### Data Acquisition

HIV-1 sequence files were downloaded (in FASTA format) from the Los Alamos National Laboratory (LANL) HIV Sequence Database (www.hiv.lanl.gov, Feb 2008). 1357 whole genome sequences (lengths between 8023 to 10280 base pairs) of various subtypes and CRF sequences deposited from year 1983 to 2005 were analyzed. To avoid database bias, several checks were carried out. Only one sequence per patient was downloaded per year in order to avoid bias within a year. Table S1A gives the number of genomes used for analysis for each year. To avoid bias in gene-specific sequence deposition, we used *Gene Cutter* (www.hiv.lanl.gov) to clip all the genes from the whole genome sequence. Thus, equal number of sequences for all genes was available for each year, which were used for the analysis. The variation in gene lengths obtained from different genomes is shown in Table S1B. The entire analysis was also done by downloading 10609 sequences available for all individual genes in the LANL Database. For SIVcpz gene sequences and HIV-1 subtype analysis, the sequences were downloaded from the LANL HIV Sequence database (www.hiv.lanl.gov).

Codon usage is known to vary between genes of an organism and is known to correlate with the expression level of the genes [5]. Housekeeping genes are expected to be codon optimized as they are required for the maintenance of basal cellular function in the cells of the organism and hence are constitutively expressed. 37 human highly expressed housekeeping genes (HHE) with average expression level greater than 5000 [36] were downloaded from GenBank [37].

### Codon Usage Analysis

The effective number of codons, GC content, and codon usage data corresponding to each gene of HIV-1 and SIVcpz were calculated using CodonW software (http://codonw.sourceforge.net). Base compositions and codon usage table for the average of human genes and Chimpanzee (*Pan troglodytes troglodytes* or *Ptt*) were retrieved from the Codon Usage Table Database (http://www.kazusa.or.jp/codon/) derived from GenBank Release 160.0 (June 15, 2007) [38]. Different scaling methods have been proposed for the study of codon usage pattern as they are associated with different biases like gene length, amino acid composition and the number of synonymous codons [39]. In order to avoid such biases in the codon usage data, we used the normalized codon frequency values calculated by scaling the frequency for each codon with respect to the maximally-used synonymous codon for a particular amino acid [40],
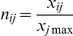
where, n_ij_ is the normalized value for i^th^ codon and j^th^ amino acid; x_ij_ is the frequency of i^th^ codon for the j^th^ amino acid and x_jmax_ is the frequency of the maximally used synonymous codon for the j^th^ amino acid. The start codon (AUG), the single codon (UGG) for tryptophan, and the three stop codons (UAA, UAG and UGA) were not included in the analysis. Table S2 gives the normalized codon usage data of HIV-1 genes extracted from the 1357 genomes along with the average codon usage data of human host.

### Statistical Analysis

*Multivariate Analysis* (MVA) is performed to reduce large number of variables or dimensions to a smaller number of new variables, so that they can be analyzed while preserving most of the information of the original data. *Factor Analysis* (FA) and *Principal Component Analysis* (PCA), are the most commonly used techniques of MVA [41]. PCA was performed on the codon usage data for the nine HIV-1 genes and the average of human genes, and FA was performed on all SIVcpz genes and *Ptt* included along with the HIV-1 genes and human. Here we had 59 variables corresponding to the 59 degenerate codons, with 10 (20 when SIVcpz and *Ptt* are included) observations corresponding to the normalized codon usage value for HIV-1 genes and human (as given in Table S2).

PCA was also performed on normalized codon usage data of gene sequences from year 1983 to 2005, with 59 variables corresponding to the degenerate codons and with 245 observations (207 observations for 9 genes, 1 for the average of human codon usage data, and 37 for the HHE genes). In the PCA, the scores for each year corresponding to a gene were studied in the hyperspace defined by the first six principal components, which accounted for 91.7% of the variance in our data set. The Euclidean metric was used as a simple deterministic measure for distance between any two points in the PCA. Here, for each year the Euclidean distance metric is calculated between the point in 6-dimensional space for each year of each gene and the average of human genes, as
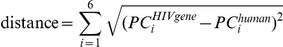
For comparative analysis, the Euclidean distance metric was normalized for each gene with respect to its maximum. The loadings of the codons for the first six principal components are given in Table S3. This analysis was performed using MATLAB R2007b (www.mathworks.com/).

The 23 years data for each gene formed a cluster of points in the PCA space. These clusters can be tight or loose depending on the variation in the codon usage pattern with time. The *Variance of a gene's cluster*, *v*, is calculated using the following formula [42],
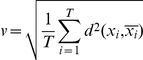
where, *T* is the number of points in the cluster, *x_i_* is the value of the *i* ^th^ point in a cluster, 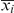 the mean value for a cluster, and *d* is the Euclidean distance metric. High or low value for *v* indicates greater or lesser variability in codon usage pattern over time respectively.

### Statistical Analysis and Clustering

The quadratic fits were computed using the MATLAB Curve Fitting Toolbox, and all statistical parameters were computed using MATLAB R2007b (www.mathworks.com/). Dendrograms were constructed using single-linkage hierarchical clustering algorithm using the MATLAB R2007b (www.mathworks.com/). The type-1 error that may occur during multiple hypotheses testing was corrected for using False Discovery Rate (FDR) method [43].

## Results

The nucleotide base composition and codon usage patterns can vary among genes of the same organism [44], and GC richness is ascribed to higher gene expression in humans [9], [45]. For the analyses, 1357 whole genome sequences of HIV-1 from different clades and geographical regions for 23 years (Table S1A) and the nine genes of HIV-1 (Table S1B) were used. We first present the study of base composition of the HIV-1 genes, to ascertain their similarity to the human host (Table S4). The patterns of codon usage of the HIV-1 genes and its host over a period of 23 years (Table S2) are then studied using Principal Component Analysis (PCA).

### Base Composition Analysis

**Figure 1:**
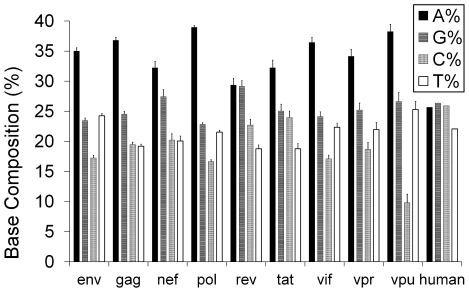
Nucleotide base content (A, G, C, and T) of HIV-1 genes and human. Error bars represent 1 standard deviation.

**Figure 2:**
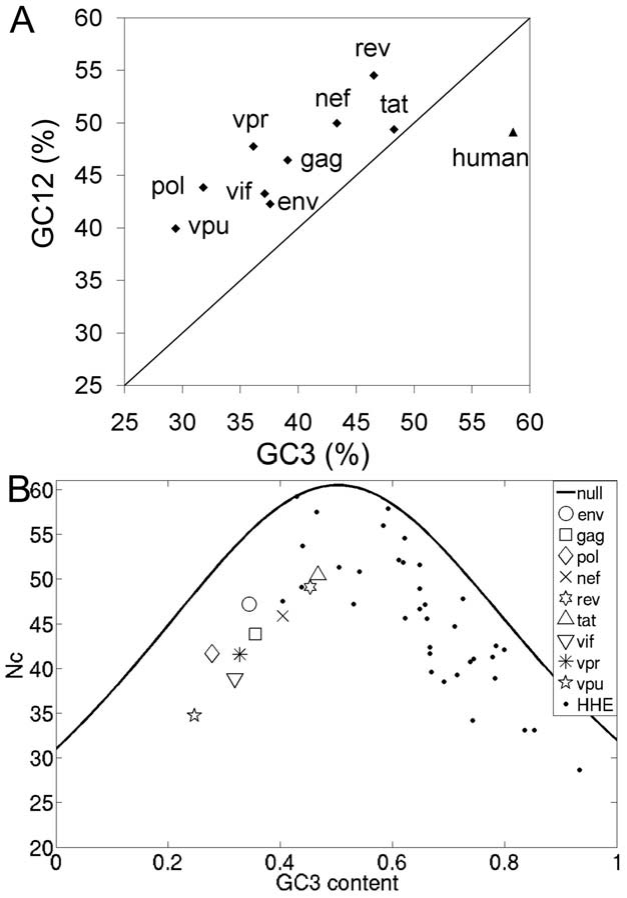
Effect of base composition on HIV-1 genes and human. (A) Relative neutrality plot and (B) Distribution of effective number of codons, Nc and GC3 content. The curve represents the null model giving the expected codon usage for a given GC3 composition. Human Highly Expressed (HHE) genes are given as filled circles.

Compared to the human genome, which has fairly uniform average base composition (approx. 22–26.5%), HIV-1 genomes are found to be A-rich (mean >36%) (Table S4). Figure 1 shows the mean base compositions of the nine HIV-1 genes, along with that of the host human. All HIV-1 genes exhibit a similar A-richness (>32%), except for *rev*, which is closer to human (25.66%). All genes, except *rev* and *tat*, have base composition distribution significantly (chi-square test, p<0.05) different from the host. The relative neutrality plot is a graph where the bases G or C at the synonymous third codon position (GC3) are plotted against the G or C content at first and second codon positions (GC12) in a gene. It gives a measure of the selective constraints acting on codons. In absence of selection, G or C content at first, second and third codon position in any gene is expected be similar, and hence lie along the diagonal in this plot. Any deviation from diagonal is indicative of selection modulating the position specific GC content (Jenkins and Holmes 2003). Figure 2A shows the relative neutrality plot for HIV-1 and average of human genes. Position specific variability in GC content in all HIV-1 genes compared to its host, which is high in GC3, is clear from this plot. This indicates a bias for A/T at the third codon position (AT3), which is even greater than the overall AT bias. In addition to this, analysis of the codon usage values show that all HIV-1 genes have more than 50% of their preferentially-used codons as A/T-ending (shown in bold in Table S2), with the structural genes (*env*, *gag*, and *pol*) and regulatory gene (*vif* and *vpu*), exhibiting extreme bias (>89%). Thus, the A/T ending codons that are rarely used by the host, are preferentially used in the late expressing genes, however, the early expressing genes (*rev*, *tat* and *nef *) exhibit lesser preference towards these rare codons. Thus, HIV-1 genomes exhibit an overall AT bias, while HIV-1 genes exhibit higher preference for A/T at the synonymous third codon position (AT3).

Codon usage can be biased by the base composition of the genes. The effective number of codons (Nc) quantifies the deviation in codon usage of a gene from the expected given the underlying base composition bias. Since, HIV-1 genes are A-rich, we plotted the effective number of codons for increasing GC3 content for the HIV-1 genes, along with a set of 37 highly expressed housekeeping genes (HHE) of the human host in Figure 2B. The null model (shown by the continuous line curve in Figure 2B) gives the expected Nc values when codon usage is determined by the base composition alone [46]. Figure 2B shows that the codon bias of all the nine HIV-1 genes is distributed and consistently much below the expected Nc values. Thus, codon bias of HIV-1 genes is not solely a result of its biased base composition. In comparison, the human highly expressed (HHE) genes are GC3 rich, and the Nc values show variable distribution with several HHE genes being closer to the expected value.

### Codon usage pattern of HIV-1 genes

**Figure 3:**
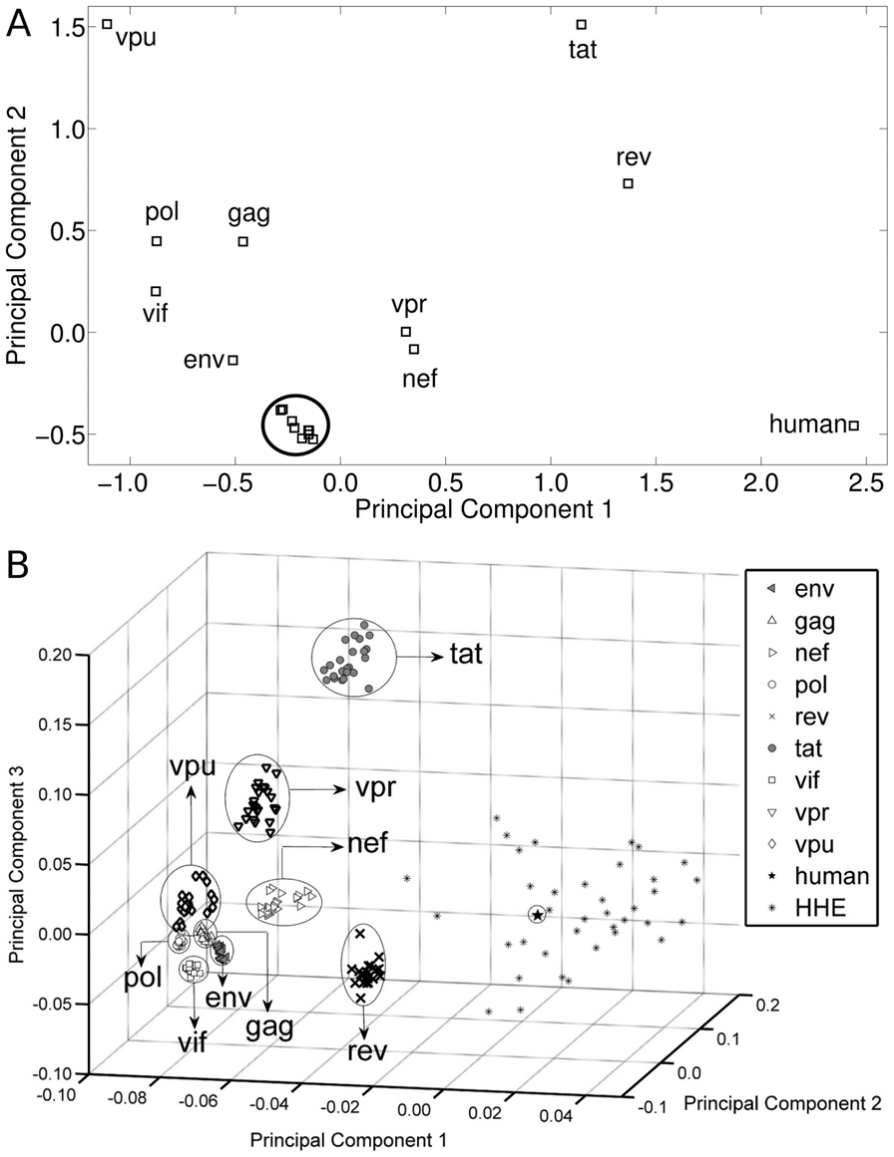
Multivariate Analysis of the codon usage pattern of nine HIV-1 genes and human. (A) First two principal component plot for nine HIV-1 genes and human (B) Three Principal Component Plot for human genes and nine HIV-1 genes for 23 years. Circles are used to distinguish the nine distinct HIV-1 gene specific clusters, with each gene specific cluster containing 23 data points corresponding to the 23 years. Here the circles are used only to mark the years corresponding to a gene. Human Highly Expressed (HHE) genes data are given as asterisks, with the average human data with the circled star.

HIV-1, a retrovirus which translates its genes using the host's cellular machinery, differs from its host in terms of base composition and codon usage pattern (Figure 1 and 2). To analyze the variation in HIV-1 genes with respect to its host, we compared the pattern of codon usage of the human genome with that of the HIV-1 genes using the 1357 whole genome sequences available in LANL database sequenced from 1983 to 2005. The codon usage pattern of nine HIV-1 genes was normalized (refer Materials and Methods) by taking the total frequency for each of the 59 codons from 1357 genes extracted from the whole genomes (Table S2) and were analyzed using PCA. The first two components (accounting for 50.3% variance) are plotted in Figure 3A. The figure shows presence of a cline in the HIV-1 genes with respect to human. Of the four regulatory genes (*nef*, *rev*, *tat* and *vpr*), *rev* gene is closest to human followed by *tat*, *nef* and *vpr*, respectively. The three structural genes (*env*, *gag*, and *pol*) and the two other regulatory genes (*vif* and *vpu*) cluster away from human. To avoid bias due to differences in gene lengths (Table S1B) in the above result, we performed PCA on 1000 random control sequences from the HIV-1 genomes for each gene. The PCA plot shows that all random control sequences cluster together (circled in Figure 3A and Supporting Information S1, Section A) and away from the human, indicating that the HIV-1 genes, in Figure 3A, exhibit a distinct non-random codon usage pattern.

### Temporal variability in the codon usage patterns of HIV-1 genes

In order to study if the non-random distribution of codon usage pattern of HIV-1 genes (Figure 3A) is solely the result of some intrinsic fluctuations, or has any temporal trend, we performed a year-wise study of the normalized codon usage pattern of the HIV-1 genes from 1983 to 2005 using PCA. Figure 3B shows the first three principal components, where points in PCA space for 23 years for a gene cluster close to each other. We circle the nine distinct HIV-1 gene-specific clusters, with each gene specific cluster containing 23 data points corresponding to the 23 years. To address whether the average human codon usage is a useful quantity to compare with the codon usage pattern of the viral genes, we included the codon usage data of the highly expressed genes (HHE), along with the average human codon usage pattern (indicated by a star). The HHE genes are calculated to be more distant from HIV-1 genes, compared to the average human codon usage data, and therefore, the average codon usage pattern is used as a fair representative for human for all other analyses. This large difference in codon usage pattern between the host and pathogen corroborates the fact that HIV-1 genes generally show low expression inside human host, and therefore codon optimization is needed for experimental studies [47]–[49].

**Figure 4:**
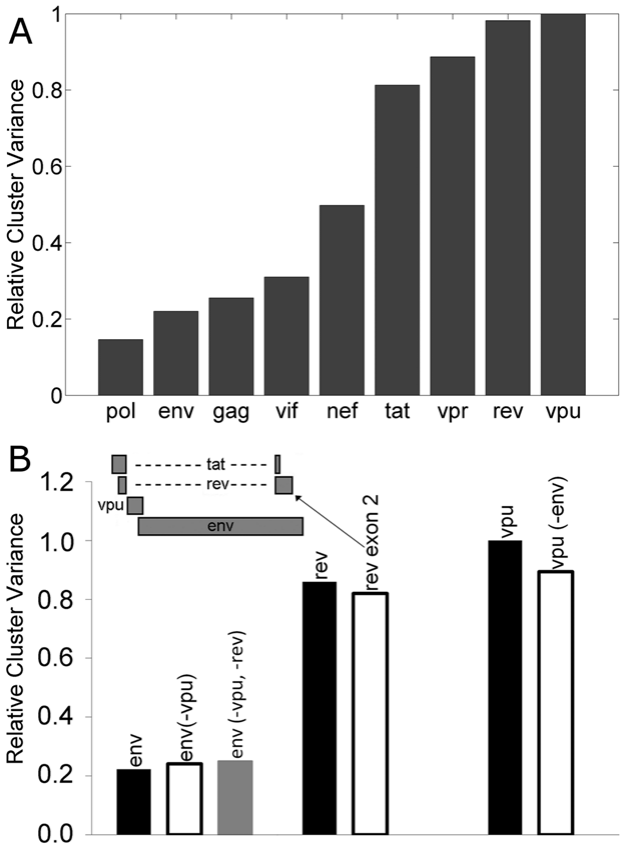
Cluster variance plots. (A) Relative cluster variance of HIV-1 genes. (B) Relative cluster variance of *env*, *rev* and *vpu* genes after removing the overlapping regions. The inset gives the genome structure of the overlapping genes.

A striking feature of Figure 3B is the variability in the cluster-compactness. HIV-1 genes show similar positional profile with respect to human in both Figures 3A and B. However, in Figure 3B, the compactness of the clusters having 23 points for each HIV-1 gene exhibits large variations. Figure 4A shows the relative cluster variances computed for each gene using the first 6 principal components (accounting for 91.7% of the variance of the original data). The four genes - *env*, *gag*, *pol* and *vif* - that clustered away from human in Figure 3A, exhibit low cluster variance over the years indicating comparatively stable codon usage pattern, while the *nef*, *rev*, *tat*, *vpr* and *vpu* genes exhibit high cluster variance indicating larger temporal variation in their codon usage pattern. Since all gene sequences for each year were obtained from the same genomes (see Data Acquisition in Materials and Methods section), this variability in Figure 4A is representative of the intrinsic difference in codon usage, and not due to within- sample variations for any specific year (see Supporting Information S1, Section A for more information).

The genome structure of HIV-1 constitutes several overlapping genes. For example, *tat*, *rev* and *vpu* overlap with the *env* gene, even though they are expressed in different frames. Since *env* showed low, whereas *tat*, *rev* and *vpu* showed large temporal variation in codon usage patterns, we compared the codon usage patterns between these overlapping genes - *env*, *rev* (second exon) and *vpu* genes (Supporting Information S1, Section B). Figure 4B shows the variance profiles of these genes, which were found to be unaltered in spite of the overlap. Thus, the genes (*nef*, *rev*, *tat*, and *vpr*) of HIV-1 not only show differential segregation (with respect to human host), they also exhibit far more temporal variability in their codon usage patterns compared to the other genes.

### Trends in Codon Usage Pattern among the HIV-1 genes

**Figure 5:**
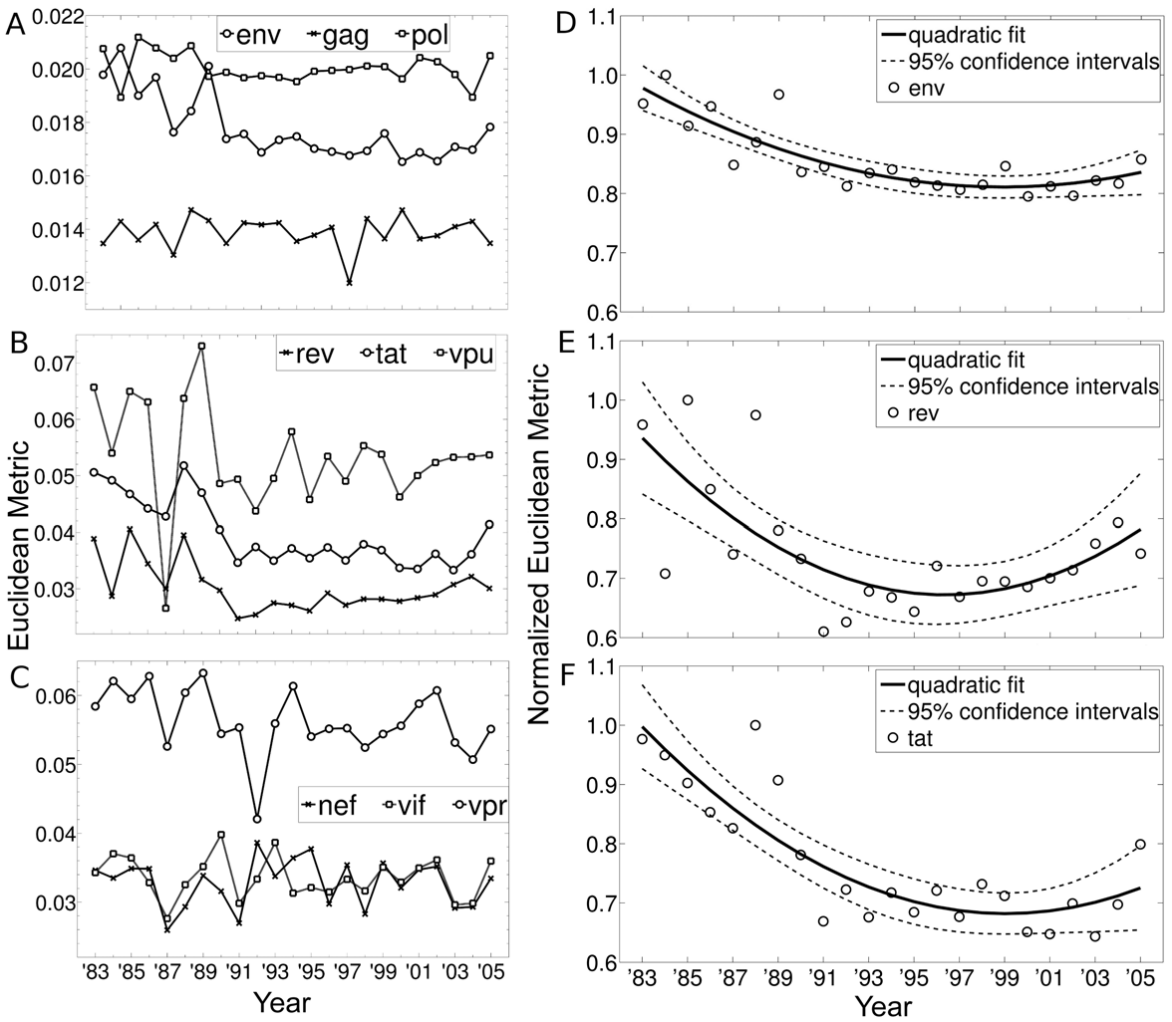
Temporal variation in the codon usage pattern of HIV-1 genes with respect to human host. The Euclidean distance metric obtained from the PCA plot for - (A) Structural genes (*env*, *gag*, and *pol*); (B) Regulatory genes (*rev*, *tat* and *vpu*); and (C) Regulatory genes (*nef*, *vif* and *vpr*). Quadratic fit of the normalized Euclidean metric data of (D) *env*, (E) *rev*, and (F) *tat*. The quadratic curves show clear negative trend for *env* (R^2^ = 0.74), *rev* (R^2^ = 0.51), and *tat* (R^2^ = 0.75) for the first 15 years.

To assess if the differential temporal variability in the codon usage patterns of the HIV-1 genes over the period of 23 years, has a specific trend or are randomly distributed, we calculated the Euclidean distance metric between each point in a cluster corresponding to each year of the HIV-1 genes with the average human data (“star”) in the PCA plot (Figure 3B), using the first 6 principal components. Figure 5A, B & C show plots for the distance metric of each HIV-1 gene from the year 1983 to 2005. From Figure 5A, B & C, two important observations can be made – (i) the structural genes (*env*, *gag*, and *pol*) exhibit low degree of variation in the distance metric with time compared to the regulatory genes; and (ii) the regulatory genes *rev*, *tat*, *vpr*, and *vpu* exhibit greater fluctuations (Figure 5B). All genes, except *nef*, exhibit a negative Kendall tau rank correlation coefficient (τ) for the first 15 years (Table S5). However, since multiple correlation analyses were performed, we corrected the result for false positives using the FDR method [43]. The three genes that exhibit high statistically significant negative temporal correlations after FDR analysis (at α = 0.05) for the first 15 years are - *rev* (τ = −0.47; p = 1.5×10^−2^), *tat* (τ = −0.62; p = 8.4×10^−4^) and *env* (τ = −0.71; p = 6.7×10^−5^). The statistical validation of the results has been shown with random control (refer Supporting Information S1, Section C and Figure S1). The two genes, *env* and *tat*, are found to exhibit low but statistically significant negative temporal correlations even if we consider all 23 years (refer Table S5). Thus, some genes of HIV-1 exhibit significant negative temporal trends, clearly indicating change in their codon usage pattern over time towards the codons preferred by the host.

For ease of comparison, we plot the Euclidean distance metric of the three genes (*env*, *rev* and *tat*) in Figure 5D, E & F after normalizing with the corresponding maximum distance value (in all 23 years). The trend in the data is shown using a quadratic fit for the three genes *env*, *rev* and *tat*. It is clear from the data that there is a clear decreasing trend for the first 15 years in all the three genes (*env*, *rev* and *tat*). However, after that there is a slight upward trend in the later years for the three genes. We have also tested the above feature using the Kullback-Leibler (KL) distance/divergence measure on the data, and obtained similar decreasing trends (result not shown). Thus, it is clear from Figure 3 and 5 that there is differential codon usage pattern among the HIV-1 genes, with *env*, *rev* and *tat* genes exhibiting increasing similarity to the human preferred codons.

The analysis shown in Figure 5D–F compares the codon usage data of the HIV-1 genes for 23 years and that of the human host's. The temporal change in codon usage pattern in some of the HIV-1 genes can also be shown without considering the human codon usage bias. Figure S2 shows the hierarchical clustering dendrogram constructed from the codon usage values for two genes using single linkage algorithm - *tat* (which exhibits a significant negative temporal correlation) and *vpr* (which does not exhibit temporal correlation). Figure S2A for the *tat* gene shows two clear clusters indicating similar codon bias - early years (1983 to 1988) and later years (1993 to 2004). The year 2005 clusters along with year 1990 for *tat* corroborating with results given in Figure 5F. However, for a gene like *vpr*, which does not exhibit any significant temporal trend, there is no clear pattern in the clustering of codon bias from early or late years (Figure S2B). Moreover, the linkage distance (Figure S2) is higher for *vpr* corroborating with its higher cluster variance compared to *tat* (as seen in Figure 4A).

In order to test if the trends observed in this population-level study are also observed in specific subtypes of HIV-1, we chose the predominant B and C subtypes of the M group prevalent in several parts of the world. Data for subtype C was available only from 1992, while for subtype B it was available from 1983. Similar analyses were performed on the codon frequencies in all genes of the subtypes B and C. The normalized Euclidean distance metrics for both subtypes B and C are shown in Table S6A and B, respectively. The structural genes in both the subtypes show little variation over the years. In comparison to Figure 5E and F, the normalized metric plots of the two early regulatory genes, *rev* and *tat*, are shown in (Figure S3) for the subtypes B and C, respectively. Here also one can observe the indications of differential trends in the codon usage patterns in *rev* and *tat* genes towards human-preferred codons. These results indicate the robustness of our analysis with all HIV-1 genomes.

### Codon Based Analysis

**Figure 6:**
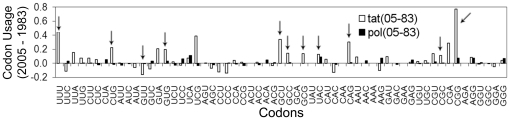
Difference between the normalized codon usage frequencies for years 2005 and 1983 for structural gene (*pol*), and regulatory gene (*tat*). Arrows indicate codons drifting towards host-preferred codons exhibiting more than 10% change.

To examine how the variation of codon usage pattern over time reflects in the usage of individual codons in HIV-1 genes, we compared the normalized frequency of each codon in each gene between the years 1983 and 2005. Due to the highly mutable nature of HIV-1, all genes show synonymous variations in more than 66% of the codons. However, more than half of these changed codons in 2005 show a shift towards the human codon usage (see Supporting Information S1, Section D). As an example, Figure 6 shows the codon-based differences between *pol*, which does not exhibit any significant trend towards human and *tat*, which exhibits significant trend towards human. The *tat* gene shows larger changes in codon frequencies (white bars), compared to the *pol* (black bars). Codons exhibiting more than 10% change are 20 for *tat*, compared to 1 for *pol*. Of the 20 codons that show more than 10% change for *tat*, 11 changed towards the host-preferred codons (shown by arrows). Clearly, codon usage pattern in *tat* gene shows a definite shift towards human-preferred codons compared to *pol*.

## Discussion

HIV-1 is a comparatively new pathogen for human. Compared to SIV that infects primates, HIV-1 has high replication rate, is highly infectious and fatal to its human host. Thus, decrypting the evolutionary trends exhibited by HIV-1, both within a particular host and on the global scale, has been an active area of research due to its relevance in the development of AIDS therapy [17]–[20], [22], [33], [34], [50], [51]. Since HIV-1 is an obligate pathogen on human for replication and assembly, codon usage bias, that affects the translational efficiency, is likely to be subjected to host selection pressure. Thus, codon usage bias can play a significant role in host adaptation of HIV-1. To our knowledge, no study has examined all the nine HIV-1 genes at the global scale over a long time period to address this issue. In this work, we have analyzed the pattern of codon usage in all genes of HIV-1 across the world for 23 years.

The two mutually non-exclusive models proposed to explain variation in codon usage are the translational selection model and the mutational model [52]. The former postulates optimization of translational efficiency/accuracy and expects codon usage co-adaptation to correlate with gene expression; whereas, the latter proposes that codon bias is a result of the genetic compositional constraints, which influence the probability of mutational fixation. Both have been shown to operate in various systems [4], [15], [53]. Differential synonymous codons usage has been shown to regulate translation in several organisms, including humans [5], [7]. Moreover, host immune response can confer strong selection pressure to shape the viral genome as has been observed in temporal studies on the evolution of influenza viruses since 1918 [54], [55].

HIV-1 genes have distinct base composition compared to its human host as shown in Figure 1. Continual selection operates on both host and pathogen to optimize virulence and also ensure survival. Being a rapidly evolving pathogen, codon usage pattern similar to host may help HIV-1 to attain higher gene expression, development of drug resistance, and immune evasion at the same time resulting in mutational robustness and translational selection [8], [13]. Most evolutionary studies on codon usage of HIV-1 have focused on either the specific codons under selection or compared the codon usage pattern of structural genes in different geographical locations [32]–[35], [51]. Our population level studies of the “Relative neutrality plot” (Figure 2A) and “Effective number of codons” (Figure 2B) of all genes of HIV-1 indicate that genetic compositional constraints may not be the primary reason for the observed codon usage bias in HIV-1 genes, and translational selection may underlie this feature. Using multivariate analysis, we show signatures of differential temporal changes in codon usage pattern in the nine genes towards the codons preferred by the host - the structural gene *env*, and two mandatory regulatory genes *rev* and *tat* expressed at the early stages of replication, are shown to become closer in terms of usage pattern to the host compared to the others. This indicates differential trends of host-specific codon adaptation in HIV-1 genes. These results, obtained through *in silico* analysis of HIV-1 genomes, can raise many questions and alternate possibilities. We discuss here a few.

Our inter-host study of all gene sequences shows that the difference in codon usage patterns for some genes of HIV-1 and the host reduces clearly for the first 15 years. As mentioned in the earlier paragraph, we can infer that sequence compositional constraint is not the only determinant factor for the observed codon usage preferences, and this may indicate a role of translation selection acting on pathogen genes, along with contribution from the ubiquitous mutational or genetic drift. A longitudinal study [32] of the codon usage pattern of *env* gene sequences from eight patients over time had shown that in 6 of the 8 patients, sequences sampled later from the patient were found to have codon usage pattern more correlated with human's compared to the sequences sampled at earlier time points. On studying the codons, Meintjes and Rodrigo [32] found that the early *env* sequences displayed a very biased codon usage pattern (possibly due to the “founder effect”), where many codons occurred at very low frequency and the preferred codons were used at a very high frequency. However, once this *homogenization* process was over, the late sequences accumulated more diversity by decreasing the usage of preferred codons and increasing the usage of codons that were not preferred over time, leading to the codon usage distribution becoming more uniform - a case of mutational drift. Such a scenario can also give rise to results similar to Figure 5 in our study. Keeping in mind that our data consists of whole genome sequences from a large number of different individuals over 23 years (and *not* sequences taken from the same patient), we compared the codon usage pattern for all the nine HIV-1 genes between the year 1983 and 2005 to identify the feature of homogenization due to genetic drift. Table S7A shows the difference between each codon for the two 6-degenerate amino acid, Arginine and Serine. The Table shows that for Serine, 26 of 54 total codons (6 codons for each of the nine genes) do not show signs of homogenization in any of the HIV-1 genes. Here, usage of 23 codons decreased in 2005 compared to 1983, in spite of the fact that they were not the preferred ones in 1983. Moreover, usage of 3 preferred codons increased in 2005 arguing against homogenization. Similarly, for Arginine (Table S7B), we find that 18 out of 54 codons argue against homogenization, where usage of 15 un-preferred codons decreased in 2005 compared to 1983 and usage of 3 preferred codons increased in 2005 compared to 1983. This closer look at the codon usages indicate that our results are not dominated by homogenization, and thus, drift is not the sole determinant of the temporal patterns observed in our analysis.

There are two facets of the temporal trend of codon usage pattern in HIV-1 genes towards its host (Figure 5D, E and F) - *fluctuations* and *negative trend*. Some genes (the smaller sized regulatory genes) are accompanied by large fluctuations (as is seen in the cluster variance plots of Figure 4A also), whereas the larger sized structural genes show lower fluctuations. It is known that smaller sequences are prone to larger stochastic variations, and much noise can be averaged out in longer sequences. However, irrespective of these fluctuations, few genes (both small and large) show statistically significant negative trend. Given what has been discussed above on the low contribution of the homogenization effect on codon usage bias, and the fact that all gene sequences arise from the same genomes (and hence are equally likely to be subjected to database bias), the random control experiment (shown in Figure 3A) indicates that the trend seen in some HIV-1 genes with respect to human codon usage pattern is an intrinsic feature of the genes, and not convergence due to stochastic effects alone.

This raises questions regarding the presence of gene-specific selective mechanisms operating on HIV-1. HIV-1 genes are regulated at more than one-step leading to optimization of the infection, as well as, the antigenic profile. The translation efficiency of *gag*-*pol* gene and *env* gene is highly enhanced in the presence of Rev protein. Earlier attempts towards development of vaccine have shown that artificial codon-optimization of *gag*, *gag*-*pol* and *env* genes lead to loss of Rev-regulated expression [47]–[49]. The codon optimization of the structural genes also leads to higher antigen presentation on the infected host cell. Higher antigen expression leads to faster detection of HIV-1 infected cells, hindering the spread of the infection. It may be noted that the negative trend in the codon usage pattern in *env* shown in our analysis, is much less compared to the regulatory genes.

Recently it was shown, using microarray data and 2D gel electrophoresis, that HIV-1 packages a variety of tRNAs including tRNA^Lys^ [56]. In addition, using Codon Adaptation Index (CAI) analysis of HXB2 reference sequences, it was shown that that the tRNA pool of human changes due to HIV-1 infection, and the early expressing genes (*rev*, *tat* and *nef *) and late expressing genes translate in different tRNA pools [56], [57]. This study suggested that the early expressing genes of HIV-1 are adapted to normal human tRNA pool, and evolved codon usage similar to those of highly expressed host genes, whereas, the late genes have evolved a different codon usage pattern. This corroborates our results in Figure 3A, which shows that the early expressing genes (*rev*, *tat* and *nef *) are closer to the human host in terms of codon usage pattern as compared to the late expressing genes. Moreover, our study also demonstrates that two of the three early expressing genes (*rev* and *tat*), which translate in normal human tRNA pool, have shown clear temporal adaptation of their codon usage pattern towards human (Figure 5 and 6). Thus, a significantly different codon biases in the genes of HIV-1 may provide a selective advantage. One may also consider the possibility that the regulatory genes, *rev* and *tat*, having a codon bias closer to the host, could prove to be effective targets to challenge HIV infection.

An issue concerns the random or upward trend in the distance metric data of the regulatory genes seen in later years in Figure 5D, E and F. Since human codon bias is considered to have remained constant over the time scale, this raises questions about the selective forces regulating the evolution of the codon bias in HIV-1 genes in terms of their fitness. This may point towards the fact that once an optimal level of codon bias is reached, increasing bias beyond this level could either maintain or reduce fitness [4]. Since we are studying changes in codon usage pattern over a short time scale (only two decades for the host), our results may represent outcome of additional and may be even contradictory selective forces (e.g., effect of anti-retroviral therapies). Also, our study calculates the codon usage pattern of the complete gene sequence, however, it is known that some regions in HIV-1 genes are more conserved compared to the others. It would be interesting to critically examine the variation in codon usage pattern in them.

SIVcpz is the predecessor of HIV-1 and infects chimpanzee (*Ptt*) in a similar manner as HIV-1 infects human [20], [21]. Since the codon usage pattern does not differ much between *Ptt* and human, and SIVcpz is known to be coexisting in *Ptt* for a very long time, what we find in Figure 3A for the HIV-1 genes may simply be a reflection of the state that already exists in SIVcpz. To test this possibility, we examined the codon usage data of the two hosts (human and *Ptt*) by doing FA with that of all the nine genes of SIVcpz and HIV-1 (Figure S4). The results indeed show the existence of a cline between *Ptt* and SIVcpz genes also, but the distances between them are considerably more, compared to that of the HIV-1 genes and human, which constitute a more recent transmission. Thus, the codon adaptation of the HIV-1 early regulatory genes towards the host's bias seems to be a feature specific to the evolution of the human-HIV-1 interaction. An interesting question is why the codon usage pattern of the SIVcpz genes, even after being in the non-human primate populations for much longer, does not move closer to the *Ptt* codon bias, as is shown by the HIV-1 genes with respect to their host. One may consider the faster replication rate, higher infectivity, and being more fatal to the host being as significant factors that can subject the HIV-1 genes to stronger selective processes in the host possibly accelerating the host-pathogen adaptive processes [17], [18], [22], [23], [28].

In summary, we have demonstrated a dynamic picture of differential host-specific adaptation in genes with different functional features, during inter-host transmission in HIV-1. Arguing against the conventional view, based primarily on studies with the structural genes, that neutral forces determine population level evolution of HIV-1, our temporal study on all genes of HIV-1 over a long period has been able to uncover statistically significant trend of host-specific adaptation in codon usage pattern in three genes of HIV-1. We show increased use of human preferred codons in HIV-1 genes indicating possible role of translation selection of this obligatory pathogen. The studies presented in this paper add to the idea that synonymous changes are not “silent” and can affect the working and evolution of the genes [9], [12], [53]. This study with codon bias analysis over time in a host pathogen system raises many questions about the subtle dynamics of synonymous changes, that alter the codon usage pattern, which remains to be studied further, and which can reveal further insights into host-pathogen co-evolution.

## Supporting Information

Figure S1 **Frequency distribution of Kendall's Tau rank correlation coefficients for -** (A) *rev* and (B) *tat* from 1983 to 1997 for 10,000 random control experiments of Figure 5E and F.
(TIF)

Figure S2 **Hierarchical clustering dendrogram constructed using single linkage algorithm on normalized codon usage values for – (A)** ***tat*** **and (B)** ***vpr*** **.** The dendrogram for *tat* show that the codon bias is distinct between early years and late years (circled) as can be seen by the clustering pattern, while the dendrogram for *vpr* does not show any pattern.
(TIF)

Figure S3 **Temporal variation in codon usage patterns with respect to host's in subtype B and C of HIV-1 for -** (A) *rev* gene: subtype B from 1987 to 2005 (linear fit, R^2^ = 0.39); subtype C from 1992 to 2005 (linear fit, R^2^ = 0.72); and (B) *tat* gene: subtype B from 1983 to 1996 (linear fit, R^2^ = 0.53) and from 1997 to 2005 (linear fit, R^2^ = 0.70); subtype C from 1992 to 2005 (linear fit, R^2^ = 0.69).
(TIF)

Figure S4 **FA bi-plot for SIV genes and Ptt (chimpanzee:** ***pan troglodytes troglodytes*** **) along with HIV-1 genes and human.**
(TIF)

Supporting Information S1 (DOC)

Table S1 (A) Yearly distribution of the 1357 HIV-1 whole genome sequences used in the study. B) Gene length range, average length, and their standard deviation of the nine HIV-1 genes extracted from the whole genomes in Table S1A. The structural genes are shaded grey.
(DOC)

Table S2 Normalized codon usage data of 18 amino acids (excluding Methionine and Tryptophan) for HIV-1 genes from all the 1357 whole genomes and human.
(DOC)

Table S3 Loadings of codons on the first six principal components used for the analysis, excluding the codons for Methionine and Tryptophan as they were not used in PCA. The loadings with absolute value >0.23 for each PC are highlighted.
(DOC)

Table S4 Nucleotide base composition of 1357 whole genome sequences of HIV-1 and average of all human genes.
(DOC)

Table S5 (A) Kendall tau rank correlation coefficient (*τ*) for the first *n* years, where *n* varies from 15 to 23 years. The significant values (p-values <0.05) of *τ* are shaded grey. (B) p-values corresponding to the Kendall tau rank correlation coefficients (*τ*) for the first n years, where n varies from 15 to 23 years. The significant p-values (<0.05) are shaded grey.
(DOC)

Table S6 A) Normalized Euclidean metric for the nine HIV-1 group M subtype B genes. The year with maximum distance for each gene is given in bold. The structural genes are shaded grey. (B) Normalized Euclidean metric for the nine HIV-1 group M subtype C genes. The year with maximum distance for each gene is given in bold. The structural genes are shaded grey.
(DOC)

Table S7 (A) Codon usage difference between the year 2005 and 1983 for 6 the serine coding codons for all the nine HIV-1 genes. Here the relative frequency of codon usage is calculated by dividing the codon usage frequency by the total number of codons used for the given year.
(DOC)

## References

[1] Krakauer DC, Jansen VAA (2002). Red queen dynamics of protein translation.. J Theor Biol.

[2] Gilchrist MA, Coombs D (2006). Evolution of virulence: Interdependence, constraints, and selection using nested models.. Theor Popul Biol.

[3] Worobey M, Bjork A, Wertheim JO (2007). Point, Counterpoint: The Evolution of Pathogenic Viruses and their Human Hosts.. Annu Rev Ecol Evol Syst.

[4] Hershberg R, Petrov DA (2008). Selection on Codon Bias.. Annu Rev Gen.

[5] Plotkin JB, Robins H, Levine AJ (2004). Tissue-specific codon usage and the expression of human genes.. Proc Natl Acad Sci USA.

[6] Rocha EP (2004). Codon usage bias from tRNA's point of view: Redundancy, specialization, and efficient decoding for translation optimization.. Genome Res.

[7] Urrutia AO, Hurst LD (2003). The signature of selection mediated by expression on human genes.. Genome Res.

[8] Wagner A (2008). Neutralism and selectionism: A network-based reconciliation.. Nat Rev Genet.

[9] Chamary JV, Parmley JL, Hurst LD (2006). Hearing silence: non-neutral evolution at synonymous sites in mammals.. Nat Rev Genet.

[10] Ikemura T (1985). Codon usage and tRNA content in unicellular and multicellular organisms.. Mol Biol Evol.

[11] Stoletzki N (2008). Conflicting selection pressures on synonymous codon use in yeast suggest selection on mRNA secondary structures.. BMC Evol Biol.

[12] Chamary JV, Hurst LD (2009). How Trivial DNA Changes Can Hurt Health.. Sci Am.

[13] Kijak GH, Currier JR, Tovanabutra S, Cox JH, Michael NL (2004). Lost in translation: implications of HIV-1 codon usage for immune escape and drug resistance.. AIDS Rev.

[14] Lucks JB, Nelson DR, Kudla GR, Plotkin JB (2008). Genome Landscapes and Bacteriophage Codon Usage.. PLoS Comput Biol.

[15] Jenkins G, Holmes E (2003). The extent of codon usage bias in human rna viruses and its evolutionary origin.. Virus Res.

[16] Jenkins GM, Pagel M, Gould EA, Zanotto PMA, Holmes EC (2001). Evolution of Base Composition and Codon Usage Bias in the Genus Flavivirus.. J Mol Evol.

[17] Rambaut A, Posada D, Crandall KA, Holmes EC (2004). The causes and consequences of HIV evolution.. Nat Rev Genet.

[18] Williamson S (2003). Adaptation in the env gene of HIV-1 and evolutionary theories of disease progression.. Mol Biol Evol.

[19] Williamson S, Perry SM, Bustamante CD, Orive ME, Stearns MN (2005). A statistical characterization of consistent patterns of human immunodeficiency virus evolution within infected patients.. Mol Biol Evol.

[20] Korber B, Muldoon M, Theiler J, Gao F, Gupta R (2000). Timing the ancestor of the HIV-1 pandemic strains.. Science.

[21] Worobey M, Gemmel M, Teuwen D, Haselkorn T, Kunstman K (2008). Direct evidence of extensive diversity of HIV-1 in Kinshasa by 1960.. Nature.

[22] Shankarappa R, Crandall KA (1999). Evolution of HIV-1 resistance to antiviral agents.. The evolution of HIV.

[23] Taylor BS, Sobieszczyk ME, McCutchan FE, Hammer SM (2008). The Challenge of HIV-1 Subtype Diversity.. N Engl J Med.

[24] Arrigo SJ, Chen IS (1991). Rev is necessary for translation but not cytoplasmic accumulation of HIV-1 vif, vpu, and env/vpu 2 RNAs.. Genes Dev.

[25] Dayton AI, Sodroski JG, Rosen CA, Goh WC, Haseltine WA (1986). The trans-activator gene of the human T cell lymphotropic virus type III is required for replication.. Cell.

[26] Fisher AG, Feinberg MB, Josephs SF, Harper ME, Marselle LM (1986). The trans-activator gene of HTLV-III is essential for virus replication.. Nature.

[27] Anderson JL, Hope TJ (2004). HIV accessory proteins and surviving the host cell.. Curr HIV/AIDS Rep.

[28] Miller RH, Sarver N (1997). HIV accessory proteins as therapeutic targets.. Nature Med.

[29] Trono D (1995). HIV accessory proteins: leading roles for the supporting cast.. Cell.

[30] Wolinsky SM, Korber BT, Neumann AU, Daniels M, Kunstman KJ, Whetsell AJ, Furtado MR, Cao Y, Ho DD, Safrit JT (1996). Adaptive evolution of human immunodeficiency virus-type 1 during the natural course of infection.. Science.

[31] Martinez MA, Vartanian JP, Wain-Hobson S (1994). Hypermutagenesis of RNA using human immunodeficiency virus type 1 reverse transcriptase and biased dNTP concentrations.. Proc Natl Acad Sci USA.

[32] Meintjes PL, Rodrigo AG (2005). Evolution of relative synonymous codon usage in Human Immunodeficiency Virus type-1.. J Bioinform Comput Biol.

[33] Choisy M, Woelk CH, Guégan JF, Robertson DL (2004). A comparative study of adaptive molecular evolution in different human immunodeficiency virus groups and subtypes.. J Virol.

[34] Woo J, Robertson DL, Lovell SC (2010). Constraints on HIV-1 diversity from protein structure.. J Virol.

[35] Ahn I, Son HS (2006). Epidemiological comparisons of codon usage patterns among HIV-1 isolates from Asia, Europe, Africa and the Americas.. Exp Mol Med.

[36] Eisenberg E, Levanon EY (2003). Human housekeeping genes are compact.. Trends Genet.

[37] Benson DA, Karsch-Mizrachi I, Lipman DJ, Ostell J, Wheeler DL (2004). Genbank: update.. Nuc Acids Res.

[38] Nakamura Y, Gojobori T, Ikemura T (2000). Codon usage tabulated from international DNA sequence databases: status for the year 2000.. Nuc Acids Res.

[39] Perriere G, Thioulouse J (2002). Use and misuse of correspondence analysis in codon usage studies.. Nuc Acids Res.

[40] Suzuki H, Saito R, Tomita M (2005). A problem in multivariate analysis of codon usage data and a possible solution.. FEBS Lett.

[41] Rencher AC (2002). Methods of Multivariate Analysis, 2nd edition.

[42] He J, Tan AH, Tan CL, Sung SY, Wu W, Xiong H, Shekhar S (2003). On quantitative evaluation of clustering systems.. Clustering and Information Retrieval.

[43] Benjamini Y, Hochberg Y (1995). Controlling the false discovery rate: a practical and powerful approach to multiple testing.. J R Statist Soc B.

[44] Grantham R, Gautier C, Gouy M, Jacobzone M, Mercier R (1981). Codon catalog usage is a genome strategy modulated for gene expressivity.. Nuc Acids Res.

[45] Muller V, Bonhoeffer S (2005). Guanine-adenine bias: a general property of retroid viruses that is unrelated to host-induced hypermutation.. Trends Genet.

[46] Wright F (1990). The ‘effective number of codons’ used in a gene.. Gene.

[47] Andre S, Seed B, Eberly J, Schraut W, Haas J (1998). Increased immune response elicited by DNA vaccination with a synthetic gp120 sequence with optimized codon usage.. J Virol.

[48] Deml L, Bojak A, Steck S, Graf M, Wild J (2001). Multiple effects of codon usage optimization on expression and immunogenicity of DNA candidate vaccines encoding the human immunodeficiency virus type-1 Gag protein.. J Virol.

[49] Kotsopoulou E, Kim VN, Kingsman AJ, Kingsman SM, Mitrophanous KA (2000). A Rev-independent human immunodeficiency virus type 1 (HIV-1)-based vector that exploits a codon-optimized HIV-1 gag-pol gene.. J Virol.

[50] Brass AL, Dykxhoorn DM, Benita Y, Yan N, Engelman A (2008). Identification of host proteins required for HIV infection through a functional genomic screen.. Science.

[51] Kosakovsky Pond SL, Frost SD, Grossman Z, Gravenor MB, Richman DD (2006). Adaptation to different human populations by HIV-1 revealed by codon-based analyses.. PLoS Comput Biol.

[52] Duret L (2002). Evolution of synonymous codon usage in metazoans.. Curr Opin Genet Devel.

[53] Plotkin JB, Kudla G (2011). Synonymous but not the same: the causes and consequences of codon bias.. Nat Rev Genet.

[54] Greenbaum BD, Levine AJ, Bhanot G, Rabadan R (2008). Patterns of Evolution and Host Gene Mimicry in Influenza and Other RNA Viruses.. PLoS Pathog.

[55] Wong EHM, Smith DK, Rabadan R, Peiris M, Poon LLM (2010). Codon usage bias and the evolution of influenza A viruses. Codon Usage Biases of Influenza Virus.. BMC Evol Biol.

[56] Pavon-Eternod M, Wei M, Pan T, Kleiman L (2010). Profiling non-lysyl tRNAs in HIV-1.. RNA.

[57] van Weringh A, Ragonnet-Cronin M, Pranckeviciene E, Pavon-Eternod M, Kleiman L (2011). HIV-1 modulates the tRNA pool to improve translation efficiency.. Mol Biol Evol.

