# Supplementary figures and images for: Differential Trends in the Codon Usage Patterns in HIV-1 Genes
Source: PLoS One. 2011 Dec 22;6(12):e28889. doi: 10.1371/journal.pone.0028889 (PMC3245234; doi:10.1371/journal.pone.0028889)

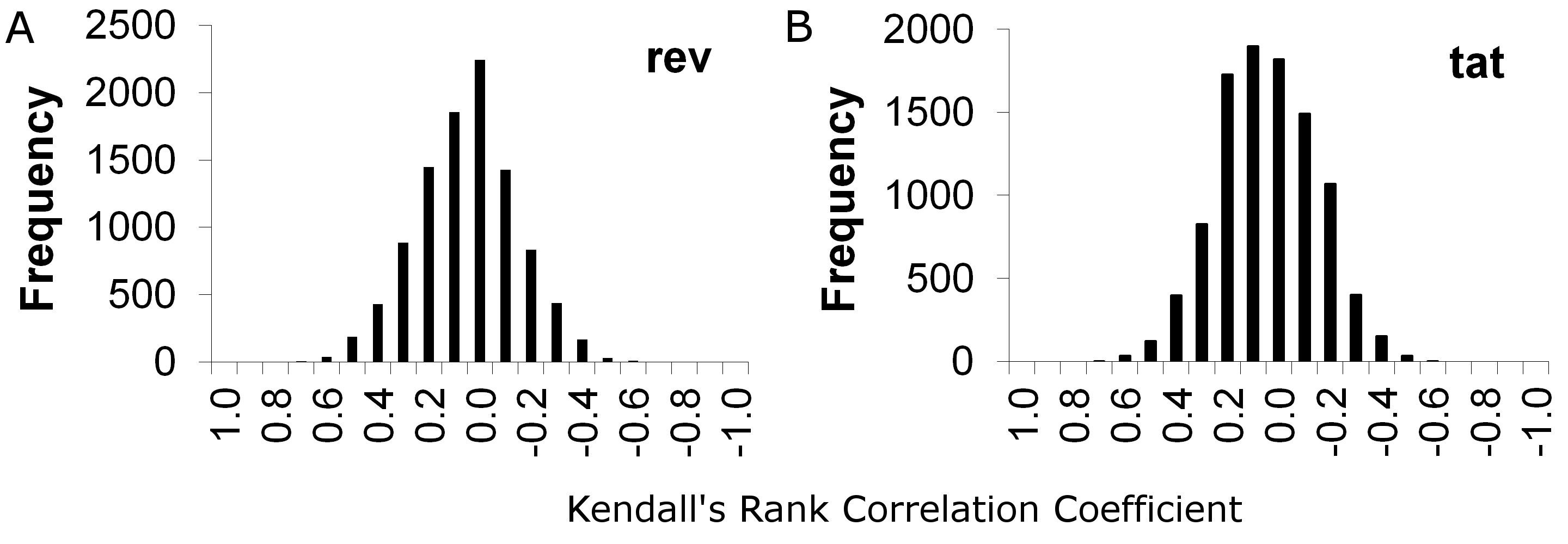

Supplement: Figure S1 — Frequency distribution of Kendall's Tau rank correlation coefficients for - (A) rev and (B) tat from 1983 to 1997 for 10,000 random control experiments of Figure 5E and F. (TIF) [file pone.0028889.s001.tif]

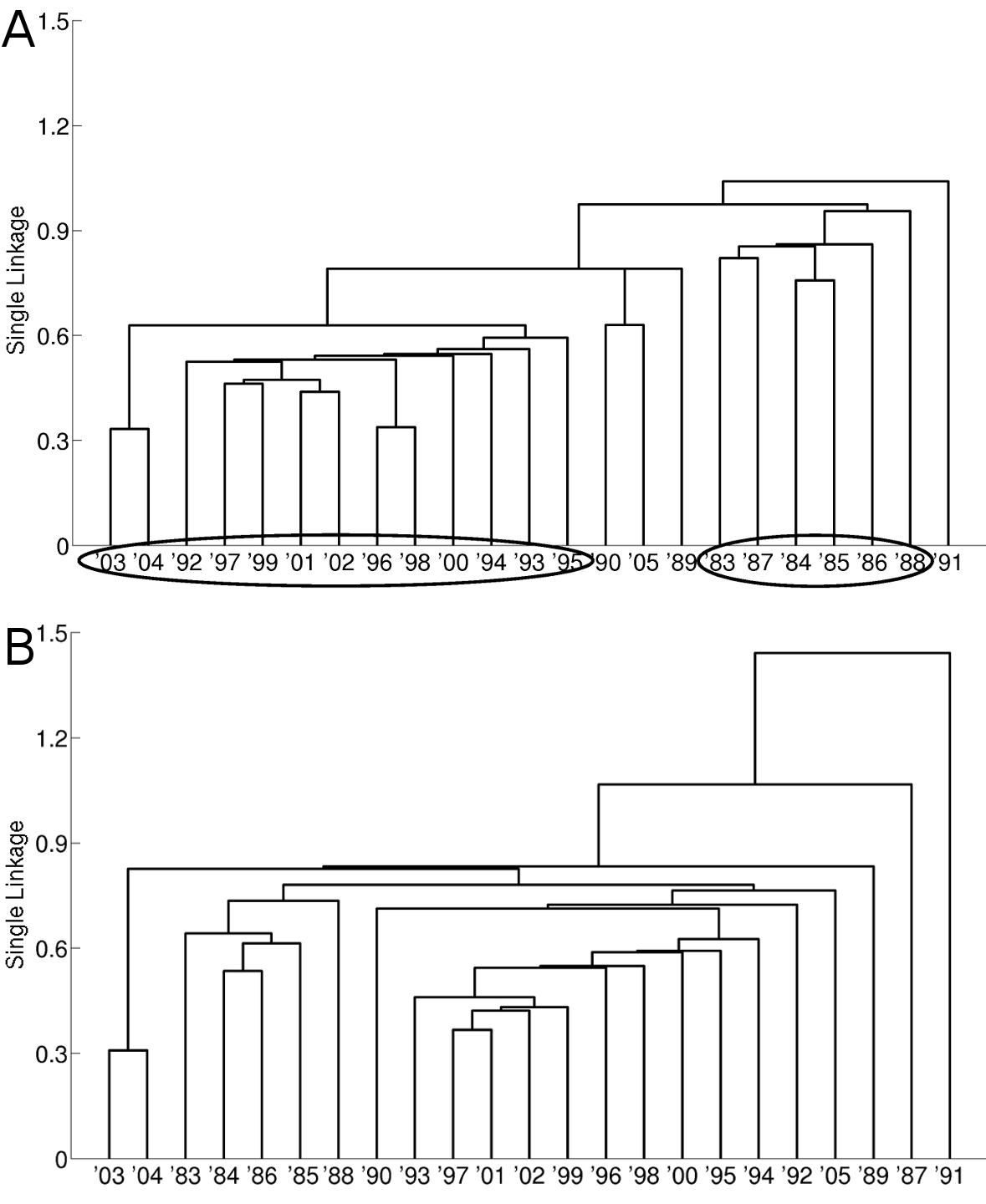

Supplement: Figure S2 — Hierarchical clustering dendrogram constructed using single linkage algorithm on normalized codon usage values for – (A) tat and (B) vpr . The dendrogram for tat show that the codon bias is distinct between early years and late years (circled) as can be seen by the clustering pattern, while the dendrogram for vpr does not show any pattern. (TIF) [file pone.0028889.s002.tif]

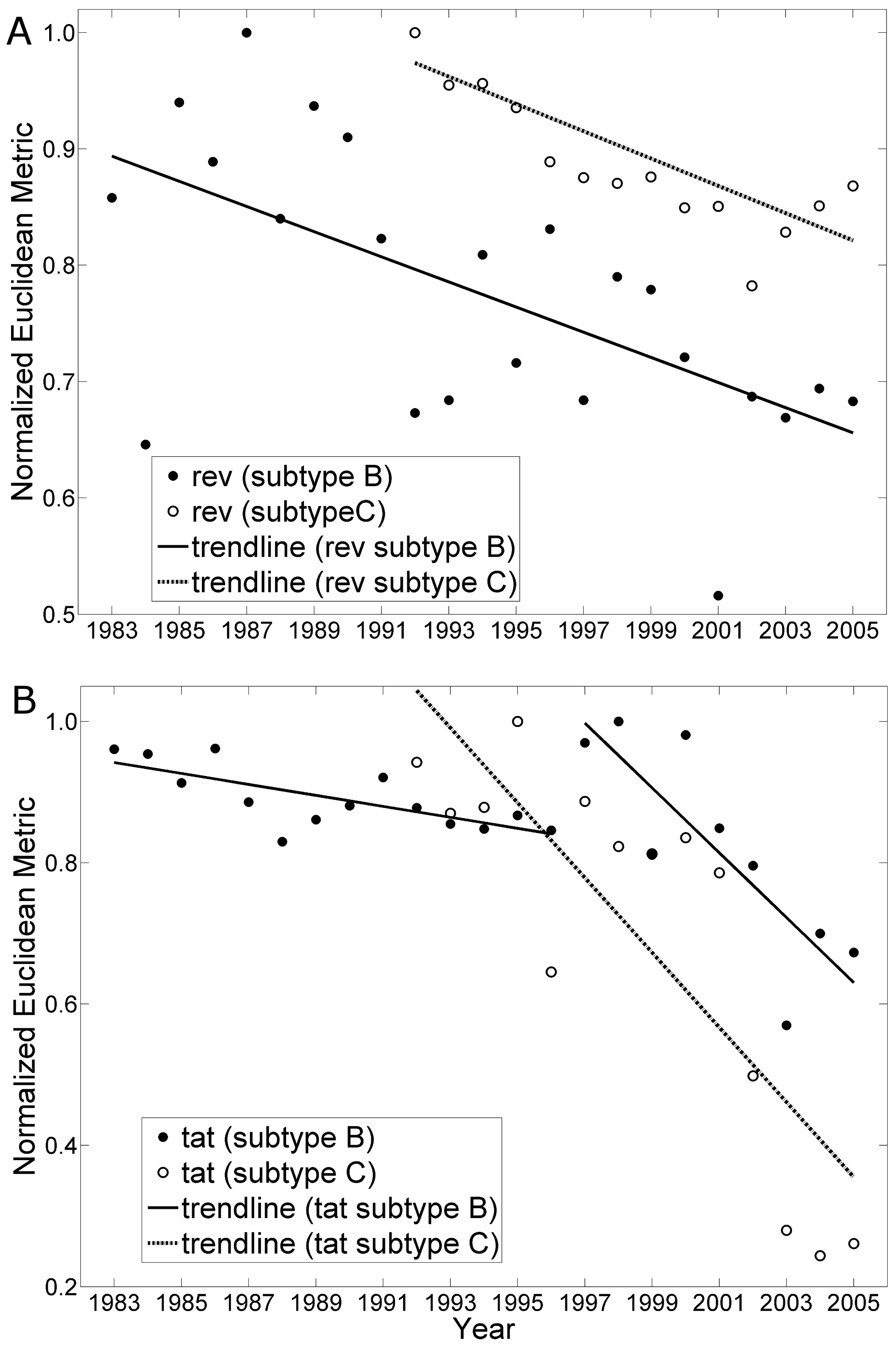

Supplement: Figure S3 — Temporal variation in codon usage patterns with respect to host's in subtype B and C of HIV-1 for - (A) rev gene: subtype B from 1987 to 2005 (linear fit, R2 = 0.39); subtype C from 1992 to 2005 (linear fit, R2 = 0.72); and (B) tat gene: subtype B from 1983 to 1996 (linear fit, R2 = 0.53) and from 1997 to 2005 (linear fit, R2 = 0.70); subtype C from 1992 to 2005 (linear fit, R2 = 0.69). (TIF) [file pone.0028889.s003.tif]

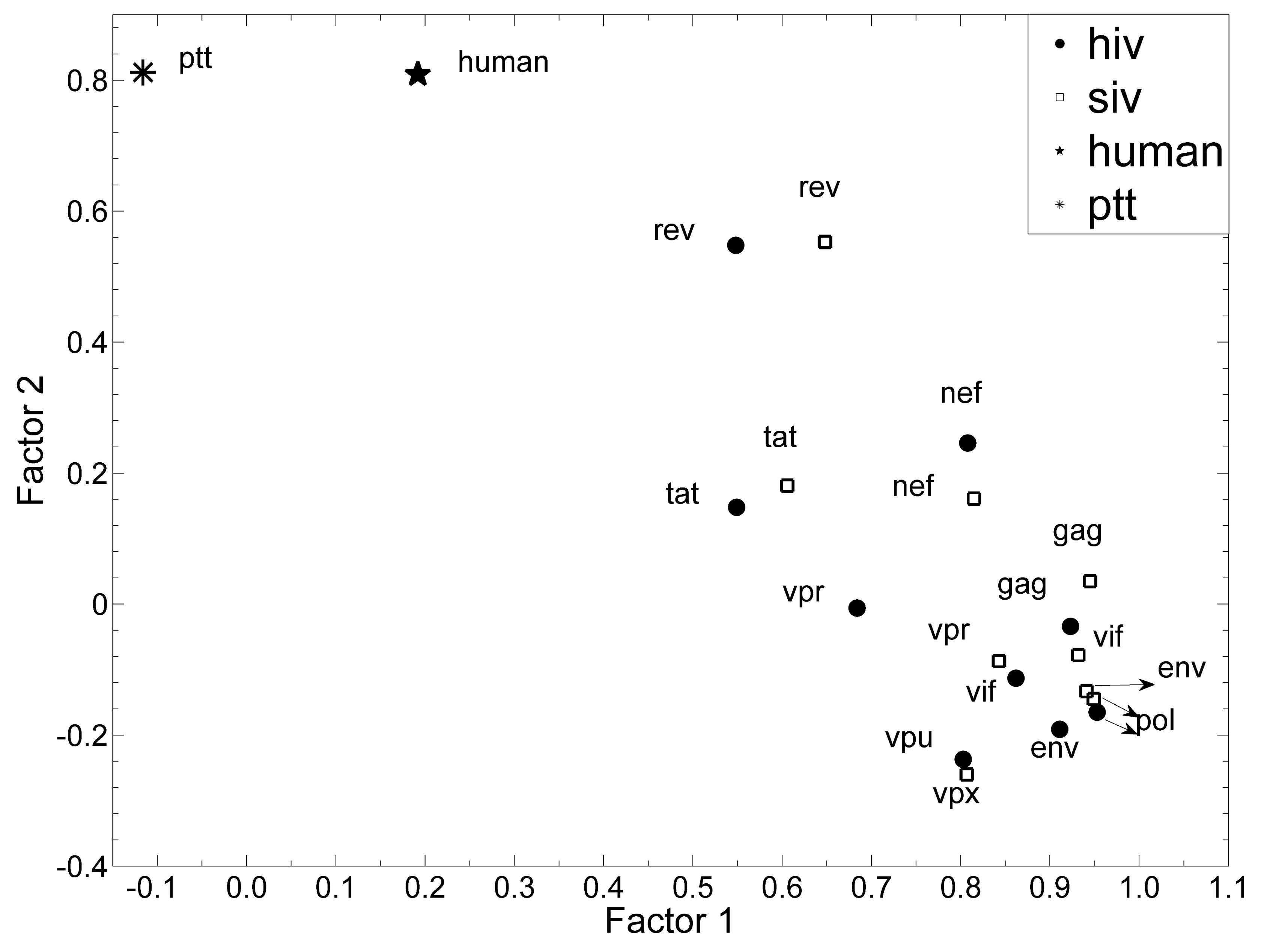

Supplement: Figure S4 — FA bi-plot for SIV genes and Ptt (chimpanzee: pan troglodytes troglodytes ) along with HIV-1 genes and human. (TIF) [file pone.0028889.s004.tif]
