# Supplementary material for: Differential Trends in the Codon Usage Patterns in HIV-1 Genes
Source: PLoS One. 2011 Dec 22;6(12):e28889. doi: 10.1371/journal.pone.0028889 (PMC3245234; doi:10.1371/journal.pone.0028889)
Supplement: Supporting Information S1 — (DOC) [file pone.0028889.s005.doc]

**SUPPORTING INFORMATION S1**

**Section A: Codon usage pattern of HIV-1 genes**

**Random control for variation in number of sequences extracted per year and gene length:**

The number of sequences deposited per year in the database varies, with more genome sequences available in the later years compared to the earlier years. To address the issue of bias in the number of sequences deposited in different years, we analyzed the data by randomly selecting 50 sequences per year for the years where the number of sequences was large. We found that variation in the number of sequences does not change the results.

To test if the codon bias varies significantly within a year we performed Kruskal-Wallis Test on the codon usage data of *tat* gene showing high cluster variance along with most significant trend. We tested for variance from year 1983, 1984 and 2005 and found no significant difference (p-value ~ 0.99) in the codon usage data of *tat* gene sequences within a year.

**Random control for variation in gene length:**

The random control experiment was done to test if the differential pattern of codon usage of the HIV-1 genes is simply a consequence of variable lengths. To test this, we randomly selected a genome from the 1357 HIV-1 whole genome sequences, and then picked up regions equivalent to the gene lengths of each gene. For each gene, 1000 iterations were carried out on which PCA was performed. Here the gene lengths are chosen from the (mean gene length ± 1 Standard Deviation) of the actual data (Table S1B). Figure 3A (inset) shows the PCA bi-plot of this data, which indicates that the pattern of codon usage in randomly picked regions of HIV-1 genome is distant from human, and independent of length of the region selected. Thus, the cline seen in the HIV-1 genes with respect to human codon usage pattern (Figure 3A) is an intrinsic feature of the genes.

**Section B: Overlap among HIV-1 genes**

The genome structure of HIV-1 constitutes several overlapping genes. For example, the regulatory genes *tat*, *rev* and *vpu* overlap with the structural gene, *env*, even though they express in a different frame. Consequently, a synonymous mutation in one gene in the overlapping region may in fact lead to a non-synonymous mutation in the other. It has been shown in SIV that a non-synonymous mutation in one gene that may occur due to selection pressure to form escape mutants, tend to induce a synonymous mutation for the other gene in the overlapping region and not change the amino acid profile of the latter [1].

To address the effect of overlapping regions in HIV-1, we checked the codon usage patterns between the *env*, *rev* (second exon) and *vpu* genes. The *vpu* gene overlaps with first 80 nucleotides of *env*, and the second exon of *rev* gene (nearly 275 nucleotides) completely overlaps with *env*. To quantify the effect of the overlapping regions between *env* and *vpu*, we removed the overlaps from both the genes for all the years and performed PCA. Figure 4B shows the cluster variances calculated for the 23 years data. It is clear that the overlapping regions did not significantly affect the variance profile of both the genes. The *vpu* non-overlapping region (white bar) exhibited similar high degree of variance as is shown by full-length *vpu* sequence (black bar). Similarly the *rev*-exon 2 (white bar), which completely overlaps with *env*, is similar to the full *rev* (black bar). Similar analysis on the *env* gene with no overlapping sequences (grey bar) shows that the overlapping regions of both the genes (*vpu* and *rev* exon 2) do not modulate the variability in codon usage pattern for the *env* gene (white and black bars).

**Section C: Kendall’s Rank Coefficient Random Control**

The negative temporal correlations for the first of 15 years (from 1983 to 1997) of *rev* and *tat* were found to be τ = -0.47 and -0.62, respectively. The correlation coefficients observed in time series analysis may be argued to result from the high order of fluctuations that appear to be an intrinsic feature of all the regulatory genes as is seen in Figure 5E and F. We, thus, randomly shuffled the codon usage data among years of each gene individually, keeping the rest as original. After shuffling, the codon usage matrix is subjected to PCA, the Euclidean distance metric of each year with respect to the host calculated, and the Kendall’s tau rank correlation coefficient (τ) for the time series for each gene measured. In Figure S1, we show the distribution of the Kendall’s correlation coefficients (τ) calculated for 10,000 iterations, from 1983 to 1997 for *rev* and *tat*. The distributions of the Kendall’s τ for the random controls clearly show that the probability of occurrence of the values, obtained from data (Figure 5E and F), by random chance is very low (p = 4 x 10^-4^ for *tat*, and p = 5.1 x 10^-3^ for *rev*). Thus, the temporal correlation coefficients obtained for *rev* and *tat* genes are highly unlikely to be due to fluctuations. It may be mentioned that a similar analysis with data from all years also shows low probability of occurrence due to chance. Given the low frequencies of occurrence, the random control eliminates the hypothesis that the high order of fluctuations seen in the genes dominates the corresponding correlation coefficients.

**Section D: Codon Based Analysis**

To quantify the codon level changes in the genes, we compare the normalized frequency of usage of each codon between the first and last year (Figure 6). The codons for which the usage frequency is enhanced in the last year compared to the first year are termed as *positively changing*, while the codons with decrease in their usage frequency are termed as *negatively changing*. We find that the number of *positively changing* codons is larger than *negatively changing* codons. We manually compared the normalized codon usage frequencies of each of the codons that vary, with that of the human codon usage frequencies, and quantified if the shift is towards human-preferred codon usage. A large proportion of the codons appear to drift towards human codon usage pattern in both structural and regulatory genes. The *vif* and *pol* codons exhibit minimum drift towards human (53.6%) while *tat* codons show the maximum drift (66.6%). However, when we examined the codons exhibiting more than 10% change, we find that relatively few codons tend to vary for the structural genes (1, 2 and 5 for *pol*, *env* and *gag* respectively) compared to the regulatory genes (8, 13, 18, 19, 19 and 20 for *vif*, *rev*, *vpu*, *nef*, *vpr* and *tat* respectively). The criterion of 10% is arbitrarily chosen by examining the variations in the codons. For all the genes, most of the positively changing codons having GC3, show increase in their frequencies and drift towards human.

**References**

1. Hughes AL, Westover K, da Silva J, O'Connor DH, Watkins DI (2001) Simultaneous positive and purifying selection on overlapping reading frames of the *tat* and *vpr* genes of simian immunodeficiency virus. J Virol 75:7966–7972.
