# Supplementary material for: Differential Trends in the Codon Usage Patterns in HIV-1 Genes
Source: PLoS One. 2011 Dec 22;6(12):e28889. doi: 10.1371/journal.pone.0028889 (PMC3245234; doi:10.1371/journal.pone.0028889)
Supplement: Table S1 — (A) Yearly distribution of the 1357 HIV-1 whole genome sequences used in the study. B) Gene length range, average length, and their standard deviation of the nine HIV-1 genes extracted from the whole genomes in Table S1A. The structural genes are shaded grey. (DOC) [file pone.0028889.s006.doc]

**Table S1: (A) Yearly distribution of the 1357 HIV-1 whole genome sequences used in the study.**

| **Year** | **Count** |
| --- | --- |
| **1983** | 14 |
| **1984** | 8 |
| **1985** | 13 |
| **1986** | 21 |
| **1987** | 5 |
| **1988** | 3 |
| **1989** | 8 |
| **1990** | 21 |
| **1991** | 6 |
| **1992** | 15 |
| **1993** | 38 |
| **1994** | 22 |
| **1995** | 23 |
| **1996** | 51 |
| **1997** | 73 |
| **1998** | 75 |
| **1999** | 152 |
| **2000** | 128 |
| **2001** | 135 |
| **2002** | 124 |
| **2003** | 195 |
| **2004** | 156 |
| **2005** | 71 |

# Table S1: (B) Gene length range, average length, and their standard deviation of the nine HIV-1 genes extracted from the whole genomes in Table S1A. The structural genes are shaded grey.

| **HIV-1**  **Genes** | **Min** | **Max** | **Average length** | **Standard Deviation** |
| --- | --- | --- | --- | --- |
| *env* | 2051 | 2731 | 2572 | 33.99 |
| *gag* | 1452 | 1566 | 1495 | 13.89 |
| *nef* | 20 | 687 | 599 | 84.76 |
| *pol* | 2940 | 3057 | 3011 | 9.72 |
| *rev* | 296 | 523 | 362 | 11.82 |
| *tat* | 251 | 323 | 306 | 2.79 |
| *vif* | 443 | 585 | 579 | 4.05 |
| *vpr* | 76 | 309 | 291 | 6.33 |
| *vpu* | 201 | 309 | 250 | 7.90 |
