# Supplementary material for: Differential Trends in the Codon Usage Patterns in HIV-1 Genes
Source: PLoS One. 2011 Dec 22;6(12):e28889. doi: 10.1371/journal.pone.0028889 (PMC3245234; doi:10.1371/journal.pone.0028889)
Supplement: Table S2 — Normalized codon usage data of 18 amino acids (excluding Methionine and Tryptophan) for HIV-1 genes from all the 1357 whole genomes and human. (DOC) [file pone.0028889.s007.doc]

**Table S2: Normalized codon usage data of 18 amino acids (excluding Methionine and Tryptophan) for HIV-1 genes from all the 1357 whole genomes and human.**

| **Amino Acid** | **Codon** | ***env*** | ***gag*** | ***nef*** | ***pol*** | ***rev*** | ***tat*** | ***vif*** | ***vpr*** | ***vpu*** | **human** |
| --- | --- | --- | --- | --- | --- | --- | --- | --- | --- | --- | --- |
| **Phe** | **UUU** | **1.000** | **1.000** | **1.000** | **1.000** | **1.000** | **1.000** | **1.000** | **1.000** | **1.000** | 0.866 |
|  | **UUC** | 0.802 | 0.653 | 0.737 | 0.532 | 0.267 | 0.903 | 0.030 | 0.603 | 0.538 | **1.000** |
| **Leu** | **UUA** | **1.000** | **1.000** | 0.937 | **1.000** | 0.055 | **1.000** | **1.000** | **1.000** | **1.000** | 0.193 |
|  | **UUG** | 0.936 | 0.341 | 0.284 | 0.289 | 0.298 | 0.080 | 0.752 | 0.330 | 0.523 | 0.326 |
|  | **CUU** | 0.570 | 0.298 | 0.379 | 0.298 | **1.000** | 0.172 | 0.016 | 0.749 | 0.417 | 0.333 |
|  | **CUC** | 0.612 | 0.252 | 0.179 | 0.189 | 0.564 | 0.056 | 0.007 | 0.466 | 0.032 | 0.494 |
|  | **CUA** | 0.772 | 0.333 | 0.776 | 0.512 | 0.290 | 0.914 | 0.835 | 0.930 | 0.274 | 0.180 |
|  | **CUG** | 0.830 | 0.213 | **1.000** | 0.300 | 0.321 | 0.189 | 0.813 | 0.959 | 0.206 | **1.000** |
| **Ile** | **AUU** | 0.527 | 0.410 | 0.598 | 0.560 | 0.780 | 0.453 | 0.555 | 0.998 | 0.218 | 0.768 |
|  | **AUC** | 0.276 | 0.284 | 0.841 | 0.281 | **1.000** | 0.940 | 0.217 | 0.425 | 0.083 | **1.000** |
|  | **AUA** | **1.000** | **1.000** | **1.000** | **1.000** | 0.309 | **1.000** | **1.000** | **1.000** | **1.000** | 0.360 |
| **Val** | **GUU** | 0.268 | 0.192 | 0.304 | 0.254 | 0.112 | 0.659 | 0.298 | **1.000** | 0.372 | 0.392 |
|  | **GUC** | 0.339 | 0.231 | 0.471 | 0.232 | 0.150 | 0.118 | 0.170 | 0.447 | 0.018 | 0.514 |
|  | **GUA** | **1.000** | **1.000** | **1.000** | **1.000** | 0.481 | **1.000** | **1.000** | 0.206 | **1.000** | 0.252 |
|  | **GUG** | 0.655 | 0.382 | 0.482 | 0.244 | **1.000** | 0.862 | 0.315 | 0.293 | 0.543 | **1.000** |
| **Ser** | **UCU** | 0.489 | 0.108 | 0.313 | 0.204 | 0.790 | 0.165 | 0.513 | 0.034 | 0.139 | 0.782 |
|  | **UCC** | 0.177 | 0.288 | 0.224 | 0.209 | 0.023 | 0.676 | 0.318 | 0.895 | 0.113 | 0.908 |
|  | **UCA** | 0.653 | 0.826 | 0.277 | 0.753 | 0.124 | 0.304 | 0.743 | 0.051 | 0.297 | 0.627 |
|  | **UCG** | 0.116 | 0.052 | 0.013 | 0.022 | 0.122 | 0.515 | 0.017 | 0.015 | 0.017 | 0.227 |
|  | **AGU** | **1.000** | 0.428 | 0.501 | **1.000** | 0.277 | 0.730 | **1.000** | 0.770 | **1.000** | 0.624 |
|  | **AGC** | 0.702 | **1.000** | **1.000** | 0.661 | **1.000** | **1.000** | 0.691 | **1.000** | 0.112 | **1.000** |
| **Pro** | **CCU** | 0.560 | 0.599 | 0.570 | 0.497 | **1.000** | **1.000** | 0.940 | 0.325 | **1.000** | 0.886 |
|  | **CCC** | 0.545 | 0.308 | 0.071 | 0.355 | 0.320 | 0.669 | 0.277 | 0.186 | 0.049 | **1.000** |
|  | **CCA** | **1.000** | **1.000** | **1.000** | **1.000** | 0.406 | 0.704 | **1.000** | **1.000** | 0.071 | 0.855 |
|  | **CCG** | 0.086 | 0.094 | 0.179 | 0.026 | 0.219 | 0.560 | 0.027 | 0.161 | 0.005 | 0.350 |
| **Thr** | **ACU** | 0.470 | 0.473 | 0.569 | 0.555 | **1.000** | 0.542 | 0.059 | **1.000** | 0.430 | 0.695 |
|  | **ACC** | 0.443 | 0.576 | 0.351 | 0.275 | 0.339 | 0.290 | 0.135 | 0.359 | 0.457 | **1.000** |
|  | **ACA** | **1.000** | **1.000** | **1.000** | **1.000** | 0.336 | **1.000** | **1.000** | 0.840 | **1.000** | 0.800 |
|  | **ACG** | 0.139 | 0.033 | 0.037 | 0.023 | 0.316 | 0.066 | 0.018 | 0.044 | 0.067 | 0.320 |
| **Ala** | **GCU** | 0.669 | 0.490 | 0.410 | 0.248 | 0.251 | **1.000** | 0.457 | 0.908 | 0.157 | 0.665 |
|  | **GCC** | 0.353 | 0.355 | 0.359 | 0.317 | 0.272 | 0.231 | 0.395 | **1.000** | 0.073 | **1.000** |
|  | **GCA** | **1.000** | **1.000** | **1.000** | **1.000** | **1.000** | 0.420 | **1.000** | 0.643 | **1.000** | 0.571 |
|  | **GCG** | 0.223 | 0.182 | 0.084 | 0.025 | 0.310 | 0.150 | 0.037 | 0.020 | 0.042 | 0.266 |
| **Tyr** | **UAU** | **1.000** | **1.000** | 0.484 | **1.000** | **1.000** | **1.000** | **1.000** | **1.000** | **1.000** | 0.796 |
|  | **UAC** | 0.416 | 0.214 | **1.000** | 0.472 | 0.862 | 0.247 | 0.222 | 0.393 | 0.079 | **1.000** |
| **His** | **CAU** | **1.000** | **1.000** | 0.887 | **1.000** | **1.000** | **1.000** | **1.000** | **1.000** | **1.000** | 0.720 |
|  | **CAC** | 0.483 | 0.690 | **1.000** | 0.491 | 0.192 | 0.181 | 0.448 | 0.167 | 0.338 | **1.000** |
| **Gln** | **CAA** | **1.000** | **1.000** | **1.000** | **1.000** | 0.444 | **1.000** | **1.000** | **1.000** | **1.000** | 0.361 |
|  | **CAG** | 0.958 | 0.843 | 0.570 | 0.735 | **1.000** | 0.883 | 0.960 | 0.634 | 0.319 | **1.000** |
| **Asn** | **AAU** | **1.000** | **1.000** | 0.784 | **1.000** | 0.944 | 0.901 | 0.618 | **1.000** | **1.000** | 0.888 |
|  | **AAC** | 0.425 | 0.631 | **1.000** | 0.368 | **1.000** | **1.000** | **1.000** | 0.207 | 0.087 | **1.000** |
| **Lys** | **AAA** | **1.000** | **1.000** | 0.982 | **1.000** | **1.000** | 0.391 | **1.000** | 0.409 | **1.000** | 0.767 |
|  | **AAG** | 0.626 | 0.565 | **1.000** | 0.440 | 0.807 | **1.000** | 0.947 | **1.000** | 0.279 | **1.000** |
| **Asp** | **GAU** | **1.000** | 0.787 | **1.000** | **1.000** | 0.595 | **1.000** | 0.582 | **1.000** | **1.000** | 0.868 |
|  | **GAC** | 0.778 | **1.000** | 0.673 | 0.683 | **1.000** | 0.330 | **1.000** | 0.746 | 0.513 | **1.000** |
| **Glu** | **GAA** | **1.000** | **1.000** | **1.000** | **1.000** | 0.486 | 0.348 | **1.000** | **1.000** | **1.000** | 0.731 |
|  | **GAG** | 0.381 | 0.605 | 0.915 | 0.431 | **1.000** | **1.000** | 0.235 | 0.409 | 0.480 | **1.000** |
| **Cys** | **UGU** | **1.000** | **1.000** | 0.505 | **1.000** | **1.000** | **1.000** | **1.000** | 0.845 | **1.000** | 0.839 |
|  | **UGC** | 0.470 | 0.288 | **1.000** | 0.200 | 0.796 | 0.863 | 0.048 | **1.000** | 0.134 | **1.000** |
| **Arg** | **CGU** | 0.019 | 0.005 | 0.039 | 0.002 | 0.042 | 0.035 | 0.018 | 0.005 | 0.006 | 0.373 |
|  | **CGC** | 0.059 | 0.006 | 0.112 | 0.008 | 0.015 | 0.087 | 0.034 | 0.003 | 0.001 | 0.856 |
|  | **CGA** | 0.058 | 0.055 | 0.173 | 0.064 | 0.475 | **1.000** | 0.015 | 0.088 | 0.004 | 0.507 |
|  | **CGG** | 0.043 | 0.095 | 0.032 | 0.057 | 0.199 | 0.398 | 0.009 | 0.016 | 0.003 | 0.939 |
|  | **AGA** | **1.000** | **1.000** | **1.000** | **1.000** | **1.000** | 0.798 | **1.000** | **1.000** | **1.000** | **1.000** |
|  | **AGG** | 0.428 | 0.642 | 0.287 | 0.499 | 0.466 | 0.493 | 0.608 | 0.289 | 0.499 | 0.983 |
| **Gly** | **GGU** | 0.275 | 0.107 | 0.133 | 0.175 | 0.086 | 0.033 | 0.289 | 0.091 | 0.165 | 0.484 |
|  | **GGC** | 0.277 | 0.431 | 0.322 | 0.103 | 0.028 | **1.000** | 0.230 | 0.395 | 0.466 | **1.000** |
|  | **GGA** | **1.000** | **1.000** | **1.000** | **1.000** | **1.000** | 0.310 | **1.000** | **1.000** | 0.642 | 0.741 |
|  | **GGG** | 0.384 | 0.643 | 0.675 | 0.522 | 0.695 | 0.252 | 0.375 | 0.950 | **1.000** | 0.741 |

Note: The preferentially used codons for each amino acid are given in bold. The structural genes are shaded grey.
