# Supplementary material for: Differential Trends in the Codon Usage Patterns in HIV-1 Genes
Source: PLoS One. 2011 Dec 22;6(12):e28889. doi: 10.1371/journal.pone.0028889 (PMC3245234; doi:10.1371/journal.pone.0028889)
Supplement: Table S3 — Loadings of codons on the first six principal components used for the analysis, excluding the codons for Methionine and Tryptophan as they were not used in PCA. The loadings with absolute value >0.23 for each PC are highlighted. (DOC) [file pone.0028889.s008.doc]

**Table S3: Loadings of codons on the first six principal components used for the analysis, excluding the codons for Methionine and Tryptophan as they were not used in PCA. The loadings with absolute value > 0.23 for each PC are highlighted.**

| **Amino Acid** | **Codon** | **PC1** | **PC2** | **PC3** | **PC4** | **PC5** | **PC6** |
| --- | --- | --- | --- | --- | --- | --- | --- |
| **Phe** | **UUU** | 0.040654 | 0.258273 | -0.20926 | -0.10214 | 0.079772 | 0.308037 |
|  | **UUC** | -0.04065 | -0.25827 | 0.209259 | 0.102142 | -0.07977 | -0.30804 |
| **Leu** | **UUA** | 0.115612 | -0.1842 | 0.047299 | -0.0355 | -0.17078 | -0.02159 |
|  | **UUG** | 0.07541 | 0.055981 | -0.0514 | 0.007058 | -0.00186 | -0.00529 |
|  | **CUU** | -0.0617 | 0.065242 | -0.00529 | 0.188713 | 0.198811 | -0.02435 |
|  | **CUC** | -0.0451 | 0.065178 | -0.01449 | 0.064687 | 0.100652 | -0.16106 |
|  | **CUA** | -0.06465 | -0.08012 | -0.07862 | -0.13592 | -0.12606 | 0.07198 |
|  | **CUG** | -0.01957 | 0.077919 | 0.102501 | -0.08903 | -0.00077 | 0.140314 |
| **Ile** | **AUU** | -0.01304 | 0.112435 | 0.025989 | -0.09138 | 0.197075 | -0.01271 |
|  | **AUC** | -0.24046 | 0.038585 | -0.07116 | 0.108548 | -0.08528 | -0.02166 |
|  | **AUA** | 0.253499 | -0.15102 | 0.045175 | -0.01717 | -0.11179 | 0.034365 |
| **Val** | **GUU** | -0.03416 | -0.16667 | 0.072408 | -0.16956 | 0.191937 | 0.163316 |
|  | **GUC** | -0.03599 | 0.055664 | 0.074125 | -0.07228 | 0.007149 | -0.06879 |
|  | **GUA** | 0.183789 | 0.056393 | -0.02935 | 0.020563 | -0.29306 | -0.10075 |
|  | **GUG** | -0.11363 | 0.054617 | -0.11718 | 0.221285 | 0.093973 | 0.006227 |
| **Ser** | **UCU** | -0.03486 | 0.111629 | -0.06624 | 0.121262 | 0.005334 | 0.023053 |
|  | **UCC** | -0.05707 | -0.13575 | 0.0322 | -0.13432 | 0.067038 | 0.059166 |
|  | **UCA** | 0.119195 | 0.036406 | -0.02115 | -0.02484 | -0.11877 | -0.19068 |
|  | **UCG** | -0.04148 | -0.04309 | -0.0501 | 0.014763 | -0.03107 | -0.03525 |
|  | **AGU** | 0.145678 | -0.10955 | 0.060369 | 0.033309 | 0.034361 | 0.212234 |
|  | **AGC** | -0.13147 | 0.140352 | 0.044913 | -0.01017 | 0.0431 | -0.06852 |
| **Pro** | **CCU** | 0.138924 | -0.07199 | -0.11599 | 0.399799 | 0.000785 | 0.397609 |
|  | **CCC** | -0.02047 | -0.01083 | -0.08487 | -0.03018 | -0.02353 | -0.23627 |
|  | **CCA** | -0.01893 | 0.128818 | 0.235886 | -0.38032 | 0.048335 | -0.15269 |
|  | **CCG** | -0.09952 | -0.04599 | -0.03502 | 0.010697 | -0.02559 | -0.00865 |
| **Thr** | **ACU** | -0.13734 | -0.00979 | 0.071928 | 0.103396 | 0.277684 | -0.02017 |
|  | **ACC** | 0.026252 | -0.01473 | 0.041886 | 0.070636 | -0.04862 | -0.14258 |
|  | **ACA** | 0.141364 | -0.01443 | -0.06912 | -0.24457 | -0.26481 | 0.19913 |
|  | **ACG** | -0.03028 | 0.038951 | -0.04469 | 0.070535 | 0.035741 | -0.03638 |
| **Ala** | **GCU** | -0.11616 | -0.19177 | -0.07194 | -0.18374 | -0.12054 | -0.05086 |
|  | **GCC** | -0.05477 | 0.022004 | 0.051366 | -0.15676 | 0.175504 | -0.00136 |
|  | **GCA** | 0.204618 | 0.12926 | 0.077076 | 0.256219 | -0.07698 | 0.143491 |
|  | **GCG** | -0.03369 | 0.040505 | -0.0565 | 0.084273 | 0.022021 | -0.09127 |
| **Tyr** | **UAU** | 0.165816 | -0.2474 | -0.32272 | -0.05725 | 0.110054 | -0.14483 |
|  | **UAC** | -0.16582 | 0.247404 | 0.32272 | 0.057254 | -0.11005 | 0.144825 |
| **His** | **CAU** | -0.05292 | -0.14614 | -0.15884 | -0.01778 | 0.233093 | 0.003761 |
|  | **CAC** | 0.052922 | 0.146144 | 0.158836 | 0.017777 | -0.23309 | -0.00376 |
| **Gln** | **CAA** | 0.099192 | -0.14708 | 0.166437 | -0.04871 | -0.0061 | 0.203445 |
|  | **CAG** | -0.09919 | 0.147079 | -0.16644 | 0.048707 | 0.006099 | -0.20344 |
| **Asn** | **AAU** | 0.15439 | -0.21016 | 0.215287 | 0.100898 | 0.285485 | -0.10682 |
|  | **AAC** | -0.15439 | 0.210164 | -0.21529 | -0.1009 | -0.28548 | 0.106822 |
| **Lys** | **AAA** | 0.2376 | 0.088285 | 0.103255 | 0.224482 | -0.11774 | -0.19052 |
|  | **AAG** | -0.2376 | -0.08828 | -0.10326 | -0.22448 | 0.11774 | 0.190523 |
| **Asp** | **GAU** | -0.02492 | -0.19541 | 0.112455 | 0.04945 | -0.08746 | 0.036029 |
|  | **GAC** | 0.024924 | 0.195412 | -0.11246 | -0.04945 | 0.08746 | -0.03603 |
| **Glu** | **GAA** | 0.26899 | 0.097188 | 0.107702 | -0.20114 | 0.087091 | 0.011853 |
|  | **GAG** | -0.26899 | -0.09719 | -0.1077 | 0.201144 | -0.08709 | -0.01185 |
| **Cys** | **UGU** | 0.268559 | 0.057995 | -0.32161 | -0.05789 | 0.12119 | -0.078 |
|  | **UGC** | -0.28114 | -0.04353 | 0.299947 | 0.028964 | -0.11054 | 0.049067 |
| **Arg** | **CGU** | -0.00876 | 0.009155 | 0.004929 | 0.003386 | -0.01394 | 0.013497 |
|  | **CGC** | -0.01595 | 0.009493 | 0.020978 | -0.00513 | -0.03475 | 0.003683 |
|  | **CGA** | -0.16431 | -0.06349 | -0.09261 | 0.062708 | -0.0562 | 0.017705 |
|  | **CGG** | -0.04761 | -0.02296 | -0.05224 | 0.024271 | -0.02928 | -0.06467 |
|  | **AGA** | 0.130826 | 0.042844 | 0.164061 | -0.0591 | 0.137187 | 0.066326 |
|  | **AGG** | 0.105811 | 0.024955 | -0.04511 | -0.02614 | -0.00301 | -0.03654 |
| **Gly** | **GGU** | 0.034571 | 0.055556 | -0.00918 | -0.03908 | -0.0347 | -0.00027 |
|  | **GGC** | -0.09119 | -0.31206 | -0.10881 | -0.04258 | -0.19187 | 0.023583 |
|  | **GGA** | 0.029926 | 0.2581 | 0.00601 | -0.0527 | 0.065589 | -0.17013 |
|  | **GGG** | 0.026695 | -0.00159 | 0.111975 | 0.134356 | 0.160981 | 0.146816 |
