# Supplementary material for: Differential Trends in the Codon Usage Patterns in HIV-1 Genes
Source: PLoS One. 2011 Dec 22;6(12):e28889. doi: 10.1371/journal.pone.0028889 (PMC3245234; doi:10.1371/journal.pone.0028889)
Supplement: Table S4 — Nucleotide base composition of 1357 whole genome sequences of HIV-1 and average of all human genes. (DOC) [file pone.0028889.s009.doc]

**Table S4: Nucleotide base composition of 1357 whole genome sequences of HIV-1 and average of all human genes.**

|  | |  | **A%** | G% | **C%** | **T%** |
| --- | --- | --- | --- | --- | --- | --- |
| **HIV** | Mean (*SD*)  Range | | 36.24 (*0.49*)  (34.56-37.61) | 23.93 (*0.30*)  (21.81-24.85) | 17.62 (*0.31*)  (16.77-19.15) | 22.16 (*0.19*)  (21.60-23.66) |
| Human | | | 25.66 | 26.35 | 25.92 | 22.07 |
