# Supplementary material for: Differential Trends in the Codon Usage Patterns in HIV-1 Genes
Source: PLoS One. 2011 Dec 22;6(12):e28889. doi: 10.1371/journal.pone.0028889 (PMC3245234; doi:10.1371/journal.pone.0028889)
Supplement: Table S5 — (A) Kendall tau rank correlation coefficient (τ) for the first n years, where n varies from 15 to 23 years. The significant values (p-values <0.05) of τ are shaded grey. (B) p-values corresponding to the Kendall tau rank correlation coefficients (τ) for the first n years, where n varies from 15 to 23 years. The significant p-values (<0.05) are shaded grey. (DOC) [file pone.0028889.s010.doc]

# Table S5: (A) Kendall tau rank correlation coefficient (*τ*) for the first *n* years, where *n* varies from 15 to 23 years. The significant values (p-values < 0.05) of *τ* are shaded grey.

| **Years** | ***env*** | ***gag*** | ***nef*** | ***pol*** | ***rev*** | ***tat*** | ***vif*** | ***vpr*** | ***vpu*** |
| --- | --- | --- | --- | --- | --- | --- | --- | --- | --- |
| 23 | -0.5178 | 0.0040 | -0.0356 | -0.0713 | -0.1067 | -0.5336 | -0.1067 | -0.2648 | -0.1462 |
| 22 | -0.6104 | 0.0694 | -0.0216 | -0.1388 | -0.1515 | -0.6190 | -0.1688 | -0.2641 | -0.1775 |
| 21 | -0.6381 | 0.0190 | 0.0381 | -0.0571 | -0.2286 | -0.6476 | -0.1048 | -0.2000 | -0.2000 |
| 20 | -0.6842 | 0.0105 | 0.1158 | -0.0316 | -0.3053 | -0.6105 | -0.0211 | -0.1474 | -0.2211 |
| 19 | -0.6608 | 0.0292 | 0.0760 | -0.0760 | -0.3567 | -0.6374 | -0.0877 | -0.2281 | -0.2398 |
| 18 | -0.6471 | 0.0719 | 0.0588 | -0.1503 | -0.3987 | -0.5948 | -0.1373 | -0.2941 | -0.2549 |
| 17 | -0.6029 | -0.0294 | 0.1029 | -0.0735 | -0.4118 | -0.5441 | -0.1471 | -0.3382 | -0.2059 |
| 16 | -0.7000 | 0.0000 | 0.0333 | -0.1167 | -0.4333 | -0.5500 | -0.2167 | -0.3167 | -0.2333 |
| 15 | -0.7143 | -0.1238 | 0.1429 | -0.1810 | -0.4667 | -0.6190 | -0.1810 | -0.2381 | -0.2952 |

**Table S5: (B) p-values corresponding to the Kendall tau rank correlation coefficients (*τ*) for the first n years, where n varies from 15 to 23 years. The significant p-values (< 0.05) are shaded grey.**

| **Years** | ***env*** | ***gag*** | ***nef*** | ***pol*** | ***rev*** | ***tat*** | ***vif*** | ***vpr*** | ***vpu*** |
| --- | --- | --- | --- | --- | --- | --- | --- | --- | --- |
| 23 | 0.0003 | 1 | 0.8346 | 0.6533 | 0.4966 | 0.0002 | 0.4966 | 0.0812 | 0.3457 |
| 22 | < 10^-04^ | 0.6722 | 0.9113 | 0.3819 | 0.3418 | < 10^-04^ | 0.2876 | 0.0908 | 0.2628 |
| 21 | < 10^-04^ | 0.9287 | 0.8347 | 0.7429 | 0.1576 | < 10^-04^ | 0.5306 | 0.2186 | 0.2186 |
| 20 | < 10^-04^ | 0.9745 | 0.5006 | 0.8728 | 0.0638 | < 10^-04^ | 0.9235 | 0.3859 | 0.1859 |
| 19 | < 10^-04^ | 0.8903 | 0.6787 | 0.6787 | 0.0344 | < 10^-04^ | 0.6288 | 0.1863 | 0.1638 |
| 18 | < 10^-04^ | 0.7089 | 0.7652 | 0.4101 | 0.0214 | 0.0003 | 0.4543 | 0.0959 | 0.1519 |
| 17 | 0.0004 | 0.9032 | 0.5976 | 0.7150 | 0.0217 | 0.0018 | 0.4397 | 0.0630 | 0.2706 |
| 16 | < 10^-04^ | 1 | 0.8944 | 0.5643 | 0.0198 | 0.0024 | 0.2651 | 0.0961 | 0.2281 |
| 15 | < 10^-04^ | 0.5589 | 0.4951 | 0.3795 | 0.0155 | 0.0008 | 0.3795 | 0.2395 | 0.1395 |
