# Supplementary material for: Differential Trends in the Codon Usage Patterns in HIV-1 Genes
Source: PLoS One. 2011 Dec 22;6(12):e28889. doi: 10.1371/journal.pone.0028889 (PMC3245234; doi:10.1371/journal.pone.0028889)
Supplement: Table S6 — A) Normalized Euclidean metric for the nine HIV-1 group M subtype B genes. The year with maximum distance for each gene is given in bold. The structural genes are shaded grey. (B) Normalized Euclidean metric for the nine HIV-1 group M subtype C genes. The year with maximum distance for each gene is given in bold. The structural genes are shaded grey. (DOC) [file pone.0028889.s011.doc]

# Table S6: (A) Normalized Euclidean metric for the nine HIV-1 group M subtype B genes. The year with maximum distance for each gene is given in bold. The structural genes are shaded grey.

| **Year** | ***env*** | ***gag*** | ***nef*** | ***pol*** | ***rev*** | ***tat*** | ***vif*** | ***vpr*** | ***vpu*** |
| --- | --- | --- | --- | --- | --- | --- | --- | --- | --- |
| **1983** | 0.932 | 0.787 | 0.949 | 0.971 | 0.858 | 0.961 | 0.974 | 0.874 | 0.824 |
| **1984** | 0.958 | 0.821 | 0.914 | 0.994 | 0.646 | 0.954 | 0.963 | 0.913 | 0.690 |
| **1985** | 0.928 | 0.873 | 0.908 | 0.932 | 0.940 | 0.913 | 0.878 | 0.902 | 0.908 |
| **1986** | 0.957 | 0.817 | 0.916 | **1.000** | 0.889 | 0.962 | 0.914 | 0.951 | **1.000** |
| **1987** | 0.938 | 0.786 | 0.929 | 0.981 | **1.000** | 0.886 | **1.000** | 0.701 | 0.841 |
| **1988** | 0.880 | 0.810 | **1.000** | 0.921 | 0.840 | 0.830 | 0.854 | 0.776 | 0.693 |
| **1989** | **1.000** | 0.819 | 0.937 | 0.984 | 0.937 | 0.861 | 0.928 | **1.000** | 0.699 |
| **1990** | 0.847 | 0.819 | 0.980 | 0.941 | 0.910 | 0.881 | 0.854 | 0.913 | 0.907 |
| **1991** | 0.963 | 0.959 | 0.929 | 0.943 | 0.823 | 0.921 | 0.964 | 0.824 | 0.803 |
| **1992** | 0.945 | 0.784 | 0.946 | 0.967 | 0.673 | 0.878 | 0.894 | 0.806 | 0.848 |
| **1993** | 0.951 | 0.735 | 0.979 | 0.959 | 0.684 | 0.855 | 0.969 | 0.722 | 0.837 |
| **1994** | 0.873 | 0.802 | 0.963 | 0.985 | 0.809 | 0.848 | 0.946 | 0.715 | 0.924 |
| **1995** | 0.851 | 0.806 | 0.886 | 0.837 | 0.716 | 0.867 | 0.948 | 0.806 | 0.836 |
| **1996** | 0.892 | 0.821 | 0.966 | 0.960 | 0.831 | 0.846 | 0.884 | 0.823 | 0.854 |
| **1997** | 0.911 | **1.000** | 0.971 | 0.941 | 0.684 | 0.970 | 0.967 | 0.777 | 0.866 |
| **1998** | 0.929 | 0.766 | 0.864 | 0.966 | 0.790 | **1.000** | 0.839 | 0.795 | 0.780 |
| **1999** | 0.873 | 0.855 | 0.905 | 0.955 | 0.779 | 0.811 | 0.923 | 0.809 | 0.764 |
| **2000** | 0.879 | 0.827 | 0.937 | 0.955 | 0.721 | 0.981 | 0.867 | 0.832 | 0.722 |
| **2001** | 0.930 | 0.849 | 0.947 | 0.996 | 0.516 | 0.849 | 0.945 | 0.806 | 0.681 |
| **2002** | 0.853 | 0.769 | 0.884 | 0.948 | 0.687 | 0.796 | 0.892 | 0.875 | 0.711 |
| **2003** | 0.860 | 0.802 | 0.925 | 0.953 | 0.669 | 0.570 | 0.955 | 0.908 | 0.869 |
| **2004** | 0.914 | 0.832 | 0.911 | 0.959 | 0.694 | 0.700 | 0.898 | 0.838 | 0.741 |
| **2005** | 0.912 | 0.799 | 0.891 | 0.938 | 0.683 | 0.673 | 0.893 | 0.826 | 0.770 |

# Table S6: (B) Normalized Euclidean metric for the nine HIV-1 group M subtype C genes. The year with maximum distance for each gene is given in bold. The structural genes are shaded grey.

| **Year** | ***env*** | ***gag*** | ***nef*** | ***pol*** | ***rev*** | ***tat*** | ***vif*** | ***vpr*** | ***vpu*** |
| --- | --- | --- | --- | --- | --- | --- | --- | --- | --- |
| **1992** | **1.0000** | 0.8076 | 0.7557 | 0.5834 | **1.0000** | 0.9425 | 0.8563 | 0.9308 | 0.7798 |
| **1993** | 0.9463 | 0.8363 | 0.9897 | **1.0000** | 0.9548 | 0.8700 | **1.0000** | 0.9762 | **1.0000** |
| **1994** | 0.9677 | 0.9040 | 0.9129 | 0.9536 | 0.9562 | 0.8785 | 0.9954 | 0.7902 | 0.9486 |
| **1995** | 0.8660 | 0.9534 | 0.5496 | 0.5701 | 0.9354 | **1.0000** | 0.9996 | 0.8670 | 0.9189 |
| **1996** | 0.9610 | 0.8965 | **1.0000** | 0.8174 | 0.8889 | 0.6454 | 0.8991 | 0.8313 | 0.8310 |
| **1997** | 0.9754 | 0.8402 | 0.9099 | 0.9282 | 0.8754 | 0.8870 | 0.9239 | **1.0000** | 0.5720 |
| **1998** | 0.9710 | 0.8573 | 0.9255 | 0.9573 | 0.8704 | 0.8231 | 0.9340 | 0.8638 | 0.8933 |
| **1999** | 0.9462 | 0.8420 | 0.9422 | 0.9189 | 0.8760 | 0.8127 | 0.9135 | 0.9196 | 0.7852 |
| **2000** | 0.9661 | 0.8709 | 0.9489 | 0.9444 | 0.8495 | 0.8356 | 0.8939 | 0.8519 | 0.8281 |
| **2001** | 0.9262 | 0.8346 | 0.9945 | 0.9411 | 0.8506 | 0.7860 | 0.9279 | 0.8092 | 0.7597 |
| **2002** | 0.9352 | **1.0000** | 0.9801 | 0.9623 | 0.7824 | 0.4984 | 0.9529 | 0.8933 | 0.7742 |
| **2003** | 0.9650 | 0.8557 | 0.9426 | 0.9383 | 0.8285 | 0.2797 | 0.8647 | 0.9005 | 0.6265 |
| **2004** | 0.9522 | 0.8662 | 0.9276 | 0.9188 | 0.8510 | 0.2439 | 0.8718 | 0.8864 | 0.7011 |
| **2005** | 0.9734 | 0.8638 | 0.9066 | 0.9705 | 0.8682 | 0.2608 | 0.9393 | 0.9139 | 0.6453 |
