# Supplementary material for: Differential Trends in the Codon Usage Patterns in HIV-1 Genes
Source: PLoS One. 2011 Dec 22;6(12):e28889. doi: 10.1371/journal.pone.0028889 (PMC3245234; doi:10.1371/journal.pone.0028889)
Supplement: Table S7 — (A) Codon usage difference between the year 2005 and 1983 for 6 the serine coding codons for all the nine HIV-1 genes. Here the relative frequency of codon usage is calculated by dividing the codon usage frequency by the total number of codons used for the given year. (DOC) [file pone.0028889.s012.doc]

# Table S7: (A) Codon usage difference between the year 2005 and 1983 for 6 the serine coding codons for all the nine HIV-1 genes. Here the relative frequency of codon usage is calculated by dividing the codon usage frequency by the total number of codons used for the given year.

|  | **env1983** | **env2005** | **Difference** |  | **pol1983** | **pol2005** | **Difference** |  | **gag1983** | **gag2005** | **Difference** |
| --- | --- | --- | --- | --- | --- | --- | --- | --- | --- | --- | --- |
| UCU | 0.140 | 0.154 | 0.014 |  | 0.087 | 0.070 | -0.017 |  | 0.039 | 0.033 | -0.006 |
| UCC | 0.080 | 0.064 | -0.015 |  | 0.060 | 0.078 | 0.018 |  | 0.083 | 0.104 | 0.021 |
| UCA | 0.204 | 0.218 | 0.014 |  | 0.232 | 0.257 | 0.026 |  | 0.341 | 0.302 | -0.039 |
| UCG | 0.036 | 0.035 | -0.001 |  | 0.021 | 0.008 | -0.013 |  | 0.041 | 0.029 | -0.012 |
| AGU | 0.341 | 0.318 | -0.024 |  | 0.395 | 0.367 | -0.028 |  | 0.122 | 0.157 | 0.035 |
| AGC | 0.199 | 0.211 | 0.012 |  | 0.205 | 0.219 | 0.014 |  | 0.373 | 0.375 | **0.002** |
|  |  |  |  |  |  |  |  |  |  |  |  |
|  | **rev1983** | **rev2005** | **Difference** |  | **tat1983** | **tat2005** | **Difference** |  | **vpu1983** | **vpu2005** | **Difference** |
| UCU | 0.225 | 0.325 | 0.100 |  | 0.116 | 0.079 | -0.037 |  | 0.113 | 0.098 | -0.016 |
| UCC | 0.020 | 0.019 | -0.001 |  | 0.368 | 0.230 | -0.138 |  | 0.132 | 0.070 | -0.062 |
| UCA | 0.086 | 0.083 | -0.003 |  | 0.116 | 0.092 | -0.024 |  | 0.283 | 0.168 | -0.115 |
| UCG | 0.046 | 0.032 | -0.014 |  | 0.084 | 0.146 | 0.062 |  | 0.000 | 0.012 | 0.012 |
| AGU | 0.139 | 0.126 | -0.013 |  | 0.263 | 0.236 | -0.027 |  | 0.358 | 0.586 | **0.227** |
| AGC | 0.483 | 0.416 | -0.067 |  | 0.053 | 0.217 | 0.164 |  | 0.113 | 0.066 | -0.047 |
|  |  |  |  |  |  |  |  |  |  |  |  |
|  | **nef1983** | **nef2005** | **Difference** |  | **vif1983** | **vif2005** | **Difference** |  | **vpr1983** | **vpr2005** | **Difference** |
| UCU | 0.124 | 0.145 | 0.021 |  | 0.142 | 0.137 | -0.005 |  | 0.000 | 0.024 | 0.024 |
| UCC | 0.103 | 0.105 | 0.002 |  | 0.087 | 0.104 | 0.017 |  | 0.226 | 0.304 | 0.078 |
| UCA | 0.097 | 0.113 | 0.016 |  | 0.230 | 0.249 | 0.019 |  | 0.016 | 0.028 | 0.012 |
| UCG | 0.000 | 0.003 | 0.003 |  | 0.000 | 0.000 | 0.000 |  | 0.065 | 0.004 | -0.061 |
| AGU | 0.221 | 0.232 | 0.012 |  | 0.301 | 0.296 | -0.004 |  | 0.339 | 0.272 | -0.067 |
| AGC | 0.455 | 0.402 | -0.053 |  | 0.240 | 0.214 | -0.027 |  | 0.355 | 0.368 | **0.013** |

Note: The cells shaded in grey indicate that the usage of codon decreased in 2005 compared to 1983, in spite of the fact that they were not the preferred ones in 1983. Codon for which the usage increased in 2005 compared to 1983, in spite of the fact that they were the preferred ones in 1983 are given in bold.

# Table S7: (B) Codon usage difference between the year 2005 and 1983 for 6 the arginine coding codons for all the nine HIV-1 genes. Here the relative frequency of codon usage is calculated by dividing the codon usage frequency by the total number of codons used for the given year.

|  | **env1983** | **env2005** | **Difference** |  | **pol1983** | **pol2005** | **Difference** |  | **gag1983** | **gag2005** | **Difference** |
| --- | --- | --- | --- | --- | --- | --- | --- | --- | --- | --- | --- |
| CGU | 0.007 | 0.009 | 0.003 |  | 0.002 | 0.001 | -0.001 |  | 0.005 | 0.002 | -0.002 |
| CGC | 0.051 | 0.039 | -0.012 |  | 0.000 | 0.003 | 0.003 |  | 0.002 | 0.003 | 0.001 |
| CGA | 0.016 | 0.026 | 0.010 |  | 0.025 | 0.036 | 0.012 |  | 0.046 | 0.029 | -0.017 |
| CGG | 0.023 | 0.029 | 0.007 |  | 0.039 | 0.036 | -0.003 |  | 0.065 | 0.060 | -0.005 |
| AGA | 0.644 | 0.628 | -0.016 |  | 0.679 | 0.637 | -0.042 |  | 0.580 | 0.572 | -0.008 |
| AGG | 0.260 | 0.268 | 0.008 |  | 0.256 | 0.286 | 0.030 |  | 0.302 | 0.333 | 0.031 |
|  |  |  |  |  |  |  |  |  |  |  |  |
|  | **rev1983** | **rev2005** | **Difference** |  | **tat1983** | **tat2005** | **Difference** |  | **vpu1983** | **vpu2005** | **Difference** |
| CGU | 0.013 | 0.021 | 0.008 |  | 0.000 | 0.009 | 0.009 |  | 0.000 | 0.000 | 0.000 |
| CGC | 0.000 | 0.014 | 0.014 |  | 0.000 | 0.012 | 0.012 |  | 0.000 | 0.000 | 0.000 |
| CGA | 0.219 | 0.222 | 0.003 |  | 0.331 | 0.332 | 0.002 |  | 0.000 | 0.004 | 0.004 |
| CGG | 0.080 | 0.076 | -0.005 |  | 0.105 | 0.129 | 0.024 |  | 0.000 | 0.006 | 0.006 |
| AGA | 0.527 | 0.454 | -0.073 |  | 0.387 | 0.336 | -0.051 |  | 0.640 | 0.686 | **0.047** |
| AGG | 0.161 | 0.212 | 0.052 |  | 0.177 | 0.182 | 0.005 |  | 0.360 | 0.303 | -0.057 |
|  |  |  |  |  |  |  |  |  |  |  |  |
|  | **nef1983** | **nef2005** | **Difference** |  | **vif1983** | **vif2005** | **Difference** |  | **vpr1983** | **vpr2005** | **Difference** |
| CGU | 0.040 | 0.026 | -0.013 |  | 0.015 | 0.013 | -0.001 |  | 0.014 | 0.003 | -0.011 |
| CGC | 0.056 | 0.060 | 0.003 |  | 0.005 | 0.012 | 0.007 |  | 0.007 | 0.008 | 0.001 |
| CGA | 0.254 | 0.147 | -0.107 |  | 0.010 | 0.013 | 0.003 |  | 0.075 | 0.049 | -0.027 |
| CGG | 0.000 | 0.012 | 0.012 |  | 0.005 | 0.009 | 0.004 |  | 0.007 | 0.018 | 0.011 |
| AGA | 0.525 | 0.559 | **0.033** |  | 0.618 | 0.609 | -0.009 |  | 0.603 | 0.696 | **0.093** |
| AGG | 0.124 | 0.195 | 0.071 |  | 0.348 | 0.344 | -0.004 |  | 0.295 | 0.228 | -0.067 |

Note: The cells shaded in grey indicate that the usage of codon decreased in 2005 compared to 1983, in spite of the fact that they were not the preferred ones in 1983. Codon for which the usage increased in 2005 compared to 1983, in spite of the fact that they were the preferred ones in 1983 are given in bold.
